# A New Dihydrochromone Dimer and Other Secondary Metabolites from Cultures of the Marine Sponge-Associated Fungi *Neosartorya fennelliae* KUFA 0811 and *Neosartorya tsunodae* KUFC 9213

**DOI:** 10.3390/md15120375

**Published:** 2017-12-01

**Authors:** Decha Kumla, Tin Shine Aung, Suradet Buttachon, Tida Dethoup, Luís Gales, José A. Pereira, Ângela Inácio, Paulo M. Costa, Michael Lee, Nazim Sekeroglu, Artur M. S. Silva, Madalena M. M. Pinto, Anake Kijjoa

**Affiliations:** 1 ICBAS-Instituto de Ciências Biomédicas Abel Salazar, Rua de Jorge Viterbo Ferreira, 228, 4050-313 Porto, Portugal; decha1987@hotmail.com (D.K.); tinshineaung@gmail.com (T.S.A.); nokrari_209@hotmail.com (S.B.); lgales@ibmc.up.pt (L.G.); jpereira@icbas.up.pt (J.A.P.); pmcosta@icbas.up.pt (P.M.C.); 2 Interdisciplinary Centre of Marine and Environmental Research (CIIMAR), Terminal de Cruzeiros do Porto de Lexões, Av. General Norton de Matos s/n, 4450-208 Matosinhos, Portugal; angelainacio@gmail.com (A.I.); madalena@ff.up.pt (M.M.M.P.); 3 Department of Plant Pathology, Faculty of Agriculture, Kasetsart University, Bangkok 10240, Thailand; tdethoup@yahoo.com; 4 Instituto de Biologia Molecular e Celular (i3S-IBMC), Universidade do Porto, Rua de Jorge Viterbo Ferreira, 228, 4050-313 Porto, Portugal; 5 Department of Chemistry, University of Leicester, University Road, Leicester LE1 7RH, UK; ml34@leicester.ac.uk; 6 Medicinal and Aromatic Plant Programme, Plant and Animal Sciences Department, Vocational School, Kilis 7 Aralık University, Kilis 79000, Turkey; nsekeroglu@gmail.com; 7 Departamento de Química & QOPNA, Universidade de Aveiro, 3810-193 Aveiro, Portugal; artur.silva@ua.pt; 8 Laboratório de Química Orgânica, Departamento de Ciências Químicas, Faculdade de Farmácia, Universidade do Porto, Rua de Jorge Viterbo Ferreira, 228, 4050-313 Porto, Portugal

**Keywords:** *Neosartorya fennelliae*, *Neosartorya tsunodae*, Trichocomaceae, dihydrochromone dimer, paecilin E, dankasterone A, chromanol derivative, marine sponge-associated fungi, antibacterial activity

## Abstract

A previously unreported dihydrochromone dimer, paecilin E (**1**), was isolated, together with eleven known compounds: β-sitostenone, ergosta-4,6,8 (14), 22-tetraen-3-one, cyathisterone, byssochlamic acid, dehydromevalonic acid lactone, chevalone B, aszonalenin, dankasterone A (**2**), helvolic acid, secalonic acid A and fellutanine A, from the culture filtrate extract of the marine sponge-associated fungus *Neosartorya fennelliae* KUFA 0811. Nine previously reported metabolites, including a chromanol derivative (**3**), (3β, 5α, 22*E*), 3,5-dihydroxyergosta-7,22-dien-6-one (**4**), byssochlamic acid, hopan-3β,22-diol, chevalone C, sartorypyrone B, helvolic acid, lumichrome and the alkaloid harmane were isolated from the culture of the marine-sponge associated fungus *Neosartorya tsunodae* KUFC 9213. Paecilin E (**1**), dankasterone A (**2**), a chromanol derivative (**3**), (3β, 5α, 22*E*)-3,5-dihydroxyergosta-7,22-dien-6-one (**4**), hopan-3β,22-diol (**5**), lumichrome (**6**), and harmane (**7**) were tested for their antibacterial activity against Gram-positive and Gram-negative reference and multidrug-resistant strains isolated from the environment. While paecilin E (**1**) was active against *S. aureus* ATCC 29213 and *E. faecalis* ATCC 29212, dankastetrone A (**2**) was only effective against *E. faecalis* ATCC 29212 and the multidrug-resistant VRE *E. faecalis* A5/102. Both compounds neither inhibit biofilm mass production in any of the strains at the concentrations tested nor exhibit synergistic association with antibiotics.

## 1. Introduction

In the past decade, marine-derived fungi have increasingly become an important source of bioactive marine natural products, since many consider them among the world’s greatest resources for unprecedented biodiversity and chemodiversity. Moreover, with established methods of cultivation, they can produce quantity of compounds with potential for medicinal chemistry development, clinical trials and marketing [1]. The fungi belonging to the genus *Neosartorya* (Trichocomaceae) have been revealed to be an important source of interesting bioactive metabolites such as polyketides, isocoumarins, ergosterol analogs, meroditerpenes, pyripyropenes, benzoic acid derivatives, prenylated indole derivatives, tryptoquivalines, fiscalins, phenylalanine-derived alkaloids and cyclopeptides [2]. Marine-derived fungi are also known to produce a myriad of structurally unique metabolites not produced by their terrestrial counterparts [3]. Our group has recently isolated and identified meroditerpene analogs and the indole alkaloids, from some marine-derived fungi from the genus *Neosartorya*, with interesting antibacterial activity against Gram-positive bacteria (*S. aureus* and *B. subtillis*) and multidrug-resistant isolates from the environment (MRSA and VRE). Some of these compounds also had synergistic effects with antibiotics to which the bacteria are resistant. Some of these compounds also inhibit biofilm formation at MIC [4].

In our ongoing search for new natural antibiotics from marine-derived fungi, we have investigated secondary metabolites from the culture of *Neosartorya fennelliae* KUFA 0811, isolated from the marine sponge *Clathria reinwardtii*, collected from Samaesan Island in the Gulf of Thailand. Previously, we only isolated two compounds from the marine sponge-associated *N. tsunodae* KUFC 9213 [5], therefore we have cultured this fungus to reexamine its secondary metabolites. 

Chromatographic fractionation and further purification of the ethyl acetate extract of *N. fennelliae* KUFA 0811, yielded a previously undescribed 2,3-dihydro-4*H*-chromen-4-one dimer which we have named paecilin E (**1**), in addition to the previously described dehydromevalonic acid lactone [6], byssochlamic acid [7], β-sitostenone [8], ergosta-4,6,8 (14), 22-tetraen-3-one [9], cyathisterone [10], dankasterone A (**2**) [11], chevalone B [12], helvolic acid [5], aszonalenin [13], secalonic acid A [14] and fellutanine A [13]. The ethyl acetate extract of *N. tsunodae* KUFC 9213 furnished, besides sartorypyrone B and helvolic acid which were previous isolated in our first study [5], byssochlamic acid [7], hopan-3β,22-diol (**5**) [15], chevalone C [16], a chromanol derivative (**3**) [17,18], (3β,5α,22*E*)-3,5-dihydroxyergosta-7,22-dien-6-one (**4**) [19], the alkaloid harmane (**7**) [20], and lumichrome (**6**) [21]. 

**Figure 1:**
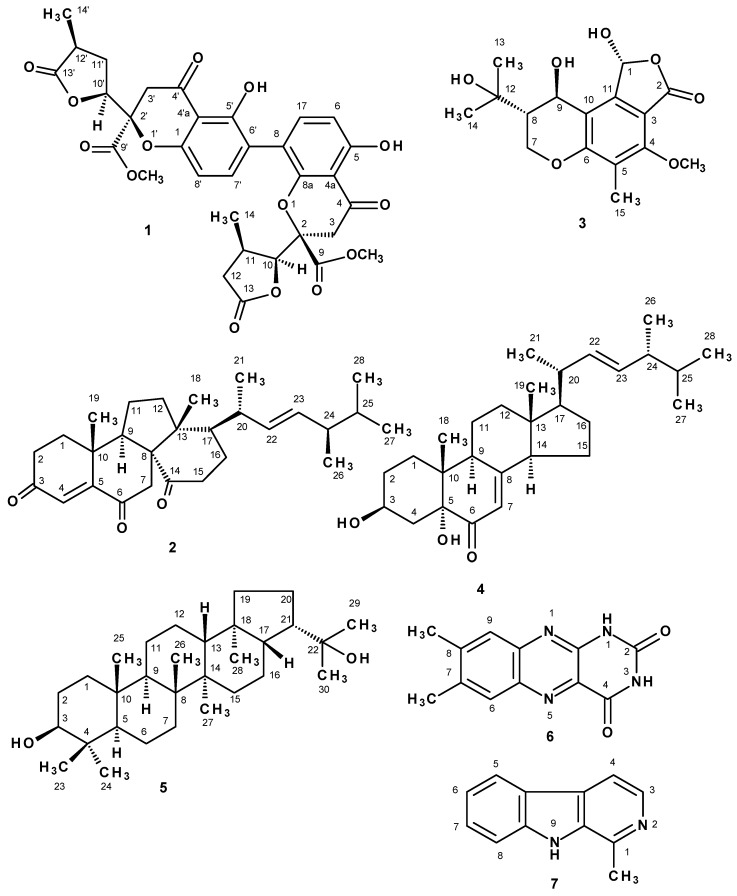
Structures of paecilin E (**1**) and dankasterone A (**2**), a chromanol derivative (**3**), (3β,5α,22*E*), 3,5-dihydroxyergosta-7,22-dien-6-one (**4**), hopan-3β,22-diol (**5**), lumichrome (**6**), harmane (**7**).

Paecilin E (**1**), dankasterone A (**2**), a chromanol derivative (**3**), (3β,5α,22E)-3,5-dihydroxyergosta-7,22-dien-6-one (**4**), hopan-3β,22-diol (**5**), lumichrome (**6**) and harmane (**7**), (Figure 1) were tested for their growth inhibitory activity against two Gram-positive (*Staphylococcus aureus* ATCC 29213 and *Enterococcus faecalis* ATCC 29212), two Gram-negative (*Escherichia coli* ATCC 25922 and *Pseudomonas aeruginosa* ATCC 27853) bacteria, a clinical isolate sensitive to the most commonly used antibiotic families, and four multidrug-resistant isolates from the environment. Paecilin E (**1**) and dankasterone A (**2**) were also investigated for their capacity to inhibit biofilm formation in the four reference strains. The potential synergism between these two compounds and the clinically used antibiotics was also investigated against multidrug-resistant isolates from the environment.

## 2. Results and Discussion

The structures of byssochlamic acid [7], hopan-3β,22-diol (**5**) [15], chevalone B [12], chevalone C [16], sartorypyrone B [5], helvolic acid [5], lumichrome (**6**) [21], harmane (**7**) [20], β-sitostenone [8], ergosta-4,6,8 [14] 22-tetraen-3-one [19], cyathisterone [10], dehydromevalonic acid lactone [6], aszonalenin [13], secalonic acid A [14] and fellutanine A [13] (Figure 1 and Supplementary Materials, Figure S1) were elucidated by analysis of their ^1^H, ^13^C NMR spectra and HRMS data, as well as by comparison of their spectral data to those reported in the literature (Supplementary Materials, Figures S2–S31).

The molecular formula of **1**, a white crystal (mp 203–205 °C), was established as C_32_H_30_O_14_ on the basis of its (+)-HRESIMS *m*/*z* 639.1718 [M + H]^+^, (calculated 639.1712 for C_32_H_31_O_14_), which indicated eighteen degrees of unsaturation. The IR spectrum showed absorption bands for hydroxyl (3443 cm^−1^), conjugated ketone carbonyl (1645 cm^−1^), ester carbonyl (1790 cm^−1^), lactone carbonyl (1738 cm^−1^), and aromatic (1470 cm^−1^) groups. The ^13^C NMR spectrum (Table 1, Supplementary Materials, Figure S33) displayed thirty two carbon signals which, based on DEPT and HSQC spectrum (Supplementary Materials, Figure S35), can be classified as two conjugated ketone carbonyl (δ_C_ 195.3 and 194.9), four ester carbonyl (δ_C_ 175.5, 174.9, 169.3 and 168.9), eight quaternary sp^2^ (δ_C_ 160.4, 158.2, 158.1, 156.1, 116.6, 114.8, 107.5 and 107.0), four methine sp^2^ (δ_C_ 140.9, 140.6, 109.2 and 107.4), two oxyquaternary sp^3^ (δ_C_ 85.4 and 83.9), two oxymethine sp^3^ (δ_C_ 81.9 and 81.7), two methoxyl (δ_C_ 53.3 and 53.3), two methine sp^3^ (δ_C_ 32.9 and 32.5), four methylene sp^3^ (δ_C_ 39.2, 39,2, 36.9, 35.3) and two methyl (δ_C_ 14.6 and 14.1) groups. Based on the type and values of their chemical shifts, these carbons were suspected to arise from two structurally similar moieties within compound **1**.

The ^1^H NMR and COSY spectra (Table 1, Supplementary Materials, Figures S32 and S34) exhibited two singlets of the hydrogen-bonded phenolic hydroxyl groups at δ_H_ 11.56 and 11.83, two pairs of *ortho*-coupled aromatic protons at δ_H_ 7.50, d (*J* = 8.6 Hz)/6.61, d (*J* = 8.6 Hz) and 7.61, d (*J* = 8.6 Hz)/6.60, d (*J* = 8.6 Hz), a pair of doublets at δ_H_ 4.85, d (*J* = 7.3 Hz) and δ_H_ 4.97, d (*J* = 6.7 Hz), two pairs of mutually coupled methylene protons at δ_H_ 3.58, d (*J* = 17.4 Hz)/3.05, d (*J* = 17.4 Hz); δ_H_ 3.57, d (*J* = 17.4 Hz)/3.09, d (*J* = 17.4 Hz) and δ_H_ 2.41, dd (*J* = 17.0, 8.4 Hz)/1.75 dd (*J* = 17.0, 9.9 Hz); δ_H_ 2.86, dd (*J* = 17.0, 8.1 Hz)/2.33, dd (*J* = 17.0, 5.4 Hz), two methyl singlets at δ_H_ 1.06, d (*J* = 7.1 Hz) and δ_H_ 1.17, d (*J* = 7.1 Hz) and two methoxyl singlets at δ_H_ 3.70 and 3.69.

The existence of a 2,2,8-trisubstituted 5-hydroxy-2,3-dihydro-4*H*-chromen-4-one moiety was substantiated by COSY correlations from H-6 (δ_H_ 6.61, d, *J* = 8.6 Hz; δ_C_ 109.2) to H-7 (δ_H_ 7.50, d, *J* = 8.6 Hz; δ_C_ 140.9), and HMBC correlations (Supplementary Materials, Figure S36) from H-6 to C-4a (δ_C_ 107.5) and C-8 (δ_C_ 114.6), H-7 to C-5 (δ_C_ 160.4) and C-8a (δ_C_ 156.1), OH-5 (δ_H_ 11.56, s) to C-4a, C-5, C-6 (δ_C_ 109.2), H-3α (δ_H_ 3.58, d, *J* = 17.4 Hz; δ_C_ 39.2) to C-2 (δ_C_ 85.4) and C-4 (δ_C_ 194.9), H-3β (δ_H_ 3.05, d, *J* = 17.4 Hz; δ_C_ 39.2) to C-4 and C-4a. One of the substituents on C-2 was deduced as a methyl formate since both H-3α and the methoxyl singlet (δ_H_ 3.70) exhibited HMBC cross peaks to the ester carbonyl at δ_C_ 168.9 (C-9). Another substituent was 4-methyldihydrofuran-2-(3*H*)-one, which linked through C-10, was substantiated by COSY correlations from H-10 (δ_H_ 4.85, d, *J* = 7.3 Hz)/H-11 (δ_H_ 2.85, m)/H_2_-12 (δ_H_ 1.75, dd, *J* = 17.0, 9.9 Hz and 2.41, dd, *J* = 17.0, 8.4 Hz), and from H-11 to Me-14 (δ_H_ 1.06, d, *J* = 7.1 Hz) as well as by HMBC correlations from H-10 to C-3, C-11 (δ_C_ 32.9), C-12 (δ_C_ 35.3) and C-13 (δ_C_ 174.9), H_2_-12 to C-10 (δ_C_ 81.7), C-13, and Me-14 (δ_C_ 14.1) as well as from H-3α to C-10. However, this first monomer constituted only half of the molecular formula, i.e., C_16_H_15_O_7_ and still lacked the substituent on C-8.

The second monomer also consisted of a 5-hydroxy-2,3-dihydro-4*H*-chromen-4-one core, but it was 2,2,6-trisubstituted as can be corroborated by COSY correlations from H-7′ (δ_H_ 7.61, d, *J* = 8.6 Hz; δ_C_ 140.6) to H-8′ (δ_H_ 6.60, d, *J* = 8.6 Hz; δ_C_ 107.4) as well as by HMBC correlations from H-7′ to C-5′ (δ_C_ 158.1), C-8′a (δ_C_ 158.2), H-8′ to C-6′ (δ_C_ 116.6) and C-4′a (δ_C_ 107.0), OH-5′ (δ_H_ 11.83, s) to C-5′, C-4′a and C-6′, H-3′β (δ_H_ 3.09, d, *J* = 17.4 Hz, δ_C_ 39.2) to C-4′ (δ_C_195.3) and C-4′a, and H-3′α (δ_H_ 3.57, dd, *J* = 17.4 Hz; δ_C_ 39.2) to C-4′ and C-2′(δ_C_ 83.9). Similarly, the substituents on C-2′ were methyl formate and 4-methyldihydrofuran-2-(3*H*)-one, through C-10′, which were based on HMBC correlations from H-3′α to C-9′ (δ_C_ 169.3), C-10′ (δ_C_ 81.9), H-10′ (δ_H_ 4.97, d, *J* = 6.4 Hz) to C-2′, C-3′, C-11′ (δ_C_ 32.5), C-13′ (δ_C_ 175.5) and Me-14′ (δ_C_ 14.6) as well as the coupling system, as observed in the COSY spectrum, from H-10′, through H-11′ (δ_H_ 2.97, m) and H_2_-12′ (δ_H_ 2.33, dd, *J* = 17.0, 5.4 Hz and 2.86, dd, *J* = 17.0, 8.1 Hz), and from H-11′ to Me-14′. Like the first monomer, the second monomer also had C_16_H_15_O_7,_ and still also lacked the substituent on C-6′. That the two monomers were connected through C-8 and C-6′ was supported by HMBC correlations from H-7 to C-6′ as well as from H-7′ to C-8. 

A literature search revealed that both monomers and dimers of 5-hydroxy-2,3-dihydro-4*H*-chromen-4-one with the methyl formate and γ-lactone substituents on C-2 have been previously reported. Guo et al. [22] reported the isolation of a 8-8′ dimer (paecilin A) and its monomer (paecilin B) of 5-hydroxy-2,3-dihydro-4*H*-chromen-4-one with the methyl formate and γ-lactone substituents on C-2 from the crude extract of mycelium of the endophytic fungus *Paeciliomyces* sp. (tree 1–7), which was isolated from mangrove bark from Xiamen, China. However, the authors did not determine the stereochemistry of both compounds. Bao et al. [23] reported the isolation, among others, of another 8-8′dimer whose ^1^H and ^13^C NMR chemical shift values of the 4-methyldihydrofuran-2-(3*H*)-one moiety were slightly different from those of paecilin A. Through the NOESY correlations, they postulated that the compound might be an epimer of paecilin A, and thus named it paecilin C. However, only the relative configurations of the stereogenic carbons of the methyl γ-lactone rings were established. El-Elimat et al. [24] mentioned the isolation of paecilin D using a bioactivity-guided fractionation of the organic extract of an unidentified fungus (MSX 45109). However, the structure of paecilin D was published later with the name 11-deoxyblennolide D [25], another monomer of 5-hydroxy-2,3-dihydro-4*H*-chromen-4-one with the methyl formate and γ-lactone substituents on C-2. 

**Figure 2:**
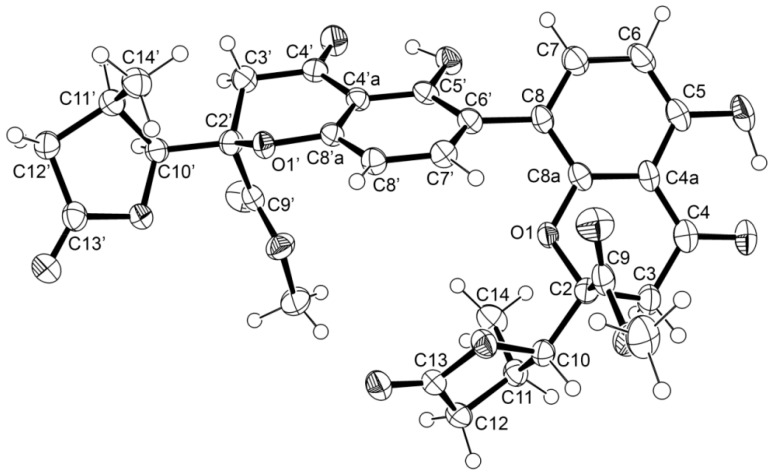
Ortep view of paecilin E (**1**).

Since **1** was obtained as a suitable crystal, its X-ray analysis was carried out. The ORTEP view, shown in Figure 2, not only confirmed the proposed structure of **1** as a 6-8 dimer of 5-hydroxy-2,3-dihydro-4*H*-chromen-4-one with the methyl formate and γ-lactone substituents on C-2, but also determined unequivocally the absolute configurations of C-2, C-2′, C-10, C-10′, C-11, C-11′ as 2*R*, 2′*R*, 10*S*, 10′*S*, 11*R* and 11′*R*. Literature search revealed that **1** has never been previously reported and therefore named paecilin E. It is worth mentioning that this is the first dimer of 5-hydroxy-2,3-dihydro-4*H*-chromen-4-one with the methyl formate and γ-lactone substituents on C-2 with complete assignment of the absolute configurations of the stereogenic carbons of both 2,3-dihydropyrone and hydroxyl-γ-lactone rings.

Analysis of the (+)-HRESIMS, ^1^H, ^13^C NMR, COSY, HSQC and HMBC and X-ray crystallographic data of compound **2** (Supplementary Materials, Table S2, Figures S37–S40 and S49) revealed that it was dankasterone A. This compound was first reported as dankasterone, a cytotoxic steroid, isolated from a marine-derived fungus *Gymnascella dankaliensis* (Castellani) Currah OUPS-N 134, by Amagata et al. [26]. However, the stereochemistry of C-24 was incorrectly assigned. Later on, Amagata and coworkers [11] published the structure of dankasterone, together with other analogs, but inverted the stereochemistry of C-24 and renamed it dankasterone A. 

Analysis of the ^1^H, ^13^C NMR, COSY, HSQC, HMBC, NOESY (Table 2, Supplementary Materials, Figures S41–S46) and (+)-HRESIMS data of **3**, revealed that it has the same planar structure as that of one of the highly substituted chromanols, isolated from cultures of *Aspergillus duricaulis* [17]. However, there were no details of the ^1^H and ^13^C NMR data of the isolated compounds. The authors have proposed that the compound was a mixture of two diastereoisomers, differing in the absolute configurations at C-1, due to a ring-chain tautomerism of the hydroxyphthalide. Moreover, the authors have found that this compound did not show any optical rotation or a Cotton effect [17] and there was no indication of the determination of the absolute configurations of any stereogenic carbons of the isolated chromanol derivatives.

**Figure 3:**
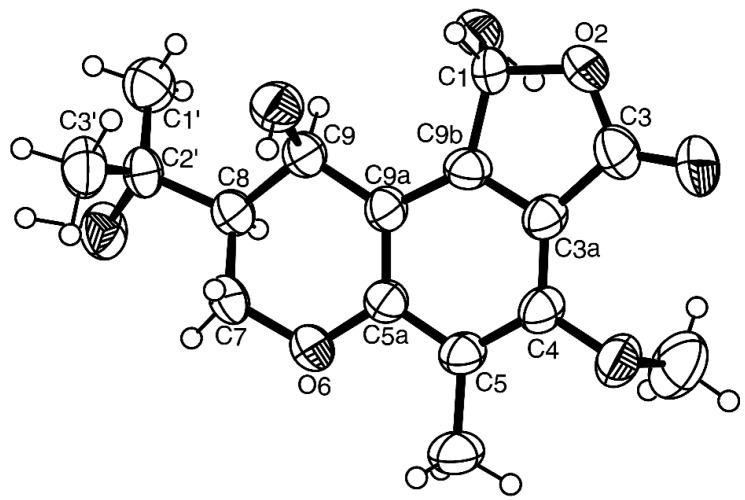
Ortep view of **3**.

Later on, the same group [18] described the same compound as colorless oil which contained a mixture of the epimers and reported two sets of ^1^H and ^13^C NMR data (in deuterated acetone) for both epimers in the mixture but without assignment of the stereochemistry of C-1. On the contrary, compound **3** is optically active (levorotatory), with [${\left[\alpha \right]}_{D}^{25}$ −80 (*c* 0.05, CHCl_3_), and exhibited only one set of the ^1^H and ^13^C NMR data (Table 2). Therefore, we concluded that **3** was a pure compound and not a mixture of the epimers as described by Archenbach et al. [17,18]. This prompted us to investigate the absolute configurations of the stereogenic carbons in **3**. Since **3** could be obtained in a suitable crystal (mp 223–224 °C), its X-ray analysis was carried out and the ORTEP view is shown in Figure 3. Therefore, **3** was identified as (1*R*, 8*S*, 9*R*)-1,9-dihydroxy-8-(2-hydroxypropan-2-yl)-4-methoxy-5-methyl-1,7,8,9-tetrahydro-3*H*-furo[3,4-f]chromen-3-one.

Analysis of the (+)-HRESIMS, ^1^H, ^13^C NMR, COSY, HSQC and HMBC data of **4** revealed that it was (3β,22*E*)-3,5-dihydroxyergosta-7,22-dien-6-one (Supplementary Materials, Table S2, Figures S47 and S48). However, from a survey of the literature, the stereochemistry of C-5 remained elusive. Aiello et al. [27] first described the isolation of 24-methylcholesta-7,22*E*-dien-3β,5α-diol-6-one and suggested that, due to the low field chemical shift of H-3 (δ_H_ 4.03, m), the hydroxyl group on C-5 was in the α position. However, no optical rotation of this compound was reported. Later on, Ishizuka et al. [28] reported the isolation of 3β,5α-dihydroxy (22*E*, 24*R*)-ergosta-7,22-dien-6-one from the fruit bodies of an edible mushroom *Grifola frondosa* (Fr.) S.F. Gray (Polyporaceae). Interestingly, the optical rotation of this compound was reported as dextrorotatory, ${\left[\alpha \right]}_{D}^{25}$ +9.1 (CHCl_3_, 0.1). Finally, the authors confirmed the structure of this compound by chemical transformation of ergosterol acetate by treatment with Na_2_Cr_2_O_7_, followed by deprotection of 3-acetoxy group. Recently, Fangkratok et al. [19] reported the isolation of (3β,5α22*E*)-3,5-dihydroxyergosta-7,22-dien-6-one from the extract of the mycelia of *Lentinus polychrous*, a Thai local edible mushroom. The ^1^H and ^13^C NMR data of this compound were very similar to those of **4** except for the chemical shift value of C-10. Furthermore, the sign of the optical rotation reported by Fangkratok et al. was levorotatory, ${\left[\alpha \right]}_{D}^{20}$ −4.37 (EtOH, 0.01), which is opposite to that of **4**, i.e., ${\left[\alpha \right]}_{D}^{20}$ +60 (CHCl_3_, 0.05). 

**Figure 4:**
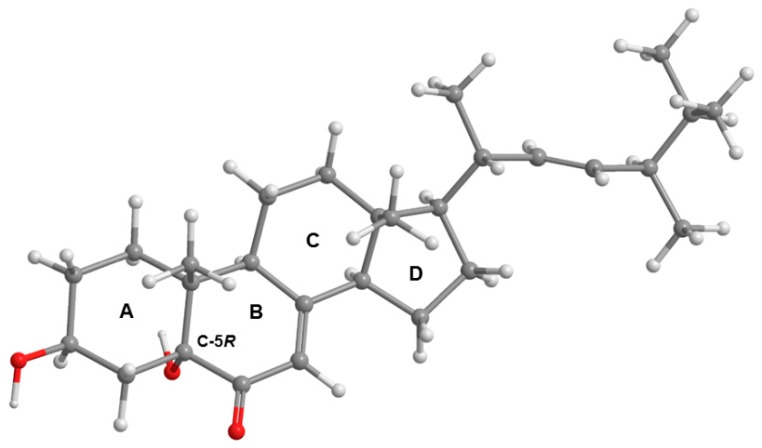
Most stable conformation of **4** (C-5*R*). Rings A and C have chair conformation.

In order to clarify the controversy and to determine unequivocally the position of the hydroxyl group on C-5 of **4**, the absolute configuration of C-5 was determined by comparison of the experimental electronic circular dichroism (ECD) spectrum with the calculated ECD spectra. Conformational analysis of the C-5*S* and C-5*R* diastereoisomers of **4** by molecular mechanics (MMFF95 force field) resulted in similar lowest energy conformations for both compounds, with rings A and C with chair conformation (Figure 4). 

**Figure 5:**
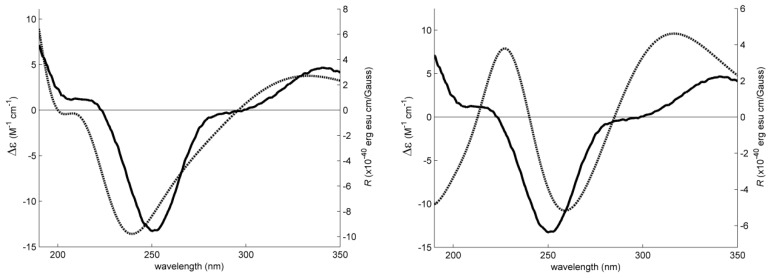
Experimental Electronic Circular Dichroism (ECD) spectrum (solid lines, **left axes**) of **4** in methanol (equal on both sides). Simulated ECD spectra (dotted lines, **right axes**) for both configurations.

However, both model’s conformational energies were further minimized by a DFT (density functional theory) method starting with ring A in chair conformation and also in boat conformation. This was considered necessary because rings A and B house the main low energy UV and ECD chromophore groups, which may engage in intramolecular hydrogen bonds, depending on the particular conformation of ring A. The DFT minimization showed that the amount of energy released by the formation of intramolecular hydrogen bonds is not enough to stabilize the boat conformations. The chair conformations are more stable than its boat counterparts in excess of 2 kcal/mol (Gibbs energy in methanol), making it overwhelmingly predominant. As such, ECD spectra were calculated for the A-chair C-5*S* and C-5*R* diastereoisomers of **4**, using a TD-DFT method. Figure 5 compares these spectra and shows how the calculated spectrum for the C-5*R* isomer fits the experimental data much better, providing enough evidence to conclude that compound **4** is the C-5*R* diastereoisomer, rather than the C-5*S*.

Paecilin E (**1**), dankasterone A (**2**), a chromanol derivative (**3**), (3β,5α,22*E*)-3,5-dihydroxyergosta-7,22-dien-6-one (**4**), hopan-3β,22-diol (**5**), lumichrome (**6**) and harmane (**7**) (Figure 1) were tested for their antibacterial activity against Gram-positive and Gram-negative bacteria, including four reference strains, a clinical isolate sensitive to the most commonly used antibiotic families, and four multidrug-resistant isolates from the environment. In the range of concentrations tested, none of the compounds were active against Gram-negative bacteria. Paecilin E (**1**) exhibited an inhibitory effect on both *Staphylococcus aureus* ATCC 29213 and *Enterococcus faecalis* ATCC 29212 (Table 3), with MIC values of 32 μg/mL and 16 μg/mL, respectively. However, when tested in a vancomycin-resistant (VRE) strain that was sensitive to ampicillin (*E. faecalis* A5/102), the MIC obtained was higher than that of the reference strain (64 μg/mL as opposed to 16 μg/mL). In the range of concentration tested, paecilin E (**1**) was ineffective against a VRE strain which was also resistant to ampicillin (*E. faecalis* B3/101). In the case of *S. aureus* strains isolated from the environment, paecilin E (**1**) was incapable of inhibiting the growth of the bacterial strain sensitive to the most commonly used antibiotic families (*S. aureus* 40/61/24) as well as of MRSA *S. aureus* 66/1. However, dankasterone A (**2**) was only effective against *E. faecalis* ATCC 29212 and VRE *E. faecalis* A5/102, with MIC of 32 μg/mL and 64 μg/mL, respectively.

The effect of paecilin E (**1**) and dankasterone A (**2**) on biofilm formation was also assessed in four reference strains and neither of them revealed an inhibitory effect on biomass production in any of the strains at the concentration tested. Regarding the screening for potential synergies between the test compounds and clinical relevant antibiotics, none of the compounds revealed a synergistic association with antibiotics, as determined by the different methodologies used. 

## 3. Experimental Section

### 3.1. General Experimental Procedures

Melting points were determined on a Bock monoscope and are uncorrected. Optical rotations were measured on an ADP410 Polarimeter (Bellingham + Stanley Ltd., Tunbridge Wells, Kent, UK). Infrared spectra were recorded in a KBr microplate in a FTIR spectrometer Nicolet iS10 from Thermo Scientific (Waltham, MA, USA) with Smart OMNI-Transmission accessory (Software 188 OMNIC 8.3). ^1^H and ^13^C NMR spectra were recorded at ambient temperature on a Bruker AMC instrument (Bruker Biosciences Corporation, Billerica, MA, USA) operating at 300 or 500 and 75 or 125 MHz, respectively. High resolution mass spectra were measured with a Waters Xevo QToF mass spectrometer (Waters Corporations, Milford, MA, USA) coupled to a Waters Aquity UPLC system. A Merck (Darmstadt, Germany) silica gel GF_254_ was used for preparative TLC, and a Merck Si gel 60 (0.2–0.5 mm) was used for column chromatography.

### 3.2. Fungal Material

The fungal strains, KUFC 9213 and KUFA 0811, were isolated from the marine sponges *Aka coralliphaga*, collected at the coral reef of Similan Islands, Phang Nga Provice (altitude 8°39′5.39″ N 97°38′16.19″ E), in April 2010 and *Clathria reinwardtii*, collected from Samaesan Island, Amphur Sattahip, Chonburi Province, Thailand (altitude 12°34′30.61″ N 100°57′5.56″ E) in February 2015, respectively. The sponge samples were washed with 0.06% sodium hypochlorite solution for 1 min, followed by sterilized seawater three times and dried on sterile filter papers under aseptic conditions. The sponges were cut into small pieces (5 × 5 mm) and placed on Petri dish plates containing 15 mL malt extract agar (MEA) medium containing 70% seawater, and incubated at 28 °C for 5–7 days. Hyphal tips emerged from sponge pieces were individually transferred onto MEA slant for further identification.

The fungi were identified to species level, based on morphological characteristics such as colony growth rate and growth pattern on standard media, namely Czapek’s agar (CZA), Czapek yeast autolysate agar (CYA), MEA and microscopic characteristics including size, shape, ornamentation of ascospores under light and scanning electron microscopes. The fungi were further identified by molecular techniques using ITS primers. DNA was extracted from young mycelia following a modified Murray and Thompson method [29]. Primer pairs ITS1 and ITS4 [30] were used for ITS gene amplification. PCR reactions were conducted on Thermal Cycler and the amplification process consisted of initial denaturation at 95 °C for 5 min, 34 cycles at 95 °C for 1 min (denaturation), at 55 °C for 1 min (annealing) and at 72 °C for 1.5 min (extension), followed by final extension at 72 °C for 10 min. PCR products were examined by Agarose gel electrophoresis (1% agarose with 1× TBE buffer) and visualized under UV light after staining with ethidium bromide. DNA sequencing analyses were sequenced using dideoxyribonucleotide chain termination method [31] by Macrogen Inc. (Seoul, Korea).

The DNA sequences were edited using FinchTV software and submitted into BLAST program for alignment and compared with that of fungal species in the NCBI database (http://www.ncbi.nlm.nih.gov/). The strain KUFC 9213 and KUFA 0811 were identified as *Neosartorya tsunodae* Yaguchi, Abliz and Y. Horie and *N. fennelliae* Kwon-Chung and S.J. Kim, respectively, and their ITS gene sequences were deposited in GenBank with accession numbers KT201524 for KUFC 9213 and KU955859 for KUFA 0811. The pure cultures were maintained at Department of Plant Pathology, Faculty of Agriculture, Kasetsart University, Bangkok, Thailand.

### 3.3. Extraction and Isolation

Each fungus was cultured for one week at 28 °C in separate Petri dish plates containing 20 mL of potato dextrose agar medium per dish. Five mycelium plugs (5 mm in diam.) of each fungus were transferred into separate 500 mL Erlenmeyer flasks containing 200 mL of potato dextrose broth and incubated on a rotary shaker at 120 rpm for one week at 28 °C to prepare mycelial suspension. Fifty 1000 mL Erlenmeyer flasks (for each fungus), each containing 300 g of cooked rice, were autoclaved at 121 °C for 15 min, and when they were cooled to room temperature, 20 mL of mycelial suspension of a fungus were inoculated per flask, and incubated at 28 °C for 30 days. Then, 500 mL of ethyl acetate was added to each moldy flask and macerated for 7 days and then filtered with Whatman No. 1 filter paper. The organic solutions were combined and evaporated under reduced pressure to furnish the crude ethyl acetate extracts of *N. tsunodae* KUFC 9213 (105 g) and *N. fennelliae* KUFA 0811 (135 g).

The crude ethyl acetate of *N. fennelliae* KUFA 0811 (135 g) was washed with H_2_O and extracted with CHCl_3_ in the same manner. The crude chloroform extract (85 g) was applied on a column of silica gel (420 g), and eluted with mixtures of petrol-CHCl_3_, CHCl_3_-Me_2_CO and CHCl_3_-MeOH, wherein 250 mL fractions were collected as follows: Frs 1–30 (petrol-CHCl_3_, 1:1), 31–86 (petrol-CHCl_3_, 3:7), 87–202 (petrol-CHCl_3_, 1:9), 203–436 (CHCl_3_), 437–579 (CHCl_3_-Me_2_CO, 9:1), 580–690 (CHCl_3_-Me_2_CO, 7:3). Frs 31–60 were combined (6.12 g) and purified by TLC (Silica gel G_254_, Petrol-CHCl_3_-EtOAc-HCO_2_H, 1:8:1:0.01) to give 16.4 mg of β-sitostenone [8] and 10.5 mg of ergosta-4,6,8 (14), 22-tetraen-3-one [9]. Frs 106–135 were combined (254 g) and purified by TLC (Silica gel G_254_, Petrol-CHCl_3_-Petrol-HCO_2_H, 9:1:0.01) to give 93 mg of yellow viscous liquid which was applied on a Sephadex LH-20 column (10 g) and eluted with MeOH and a 1:1 mixture of MeOH:CH_2_Cl_2_ wherein 1 mL 30 sfrs were collected. Sfrs 16–30 were combined and crystallized in a mixture of CHCl3 and MeOH to give 12.5 mg of dehydromevalonic acid lactone [6]. Frs 211–255 were combined (201 mg) and crystalized in a mixture of CHCl_3_ and petrol to give 12.3 mg of byssochlamic acid. The mother liquor was combined with the combined frs 136–165 (546 mg) and the combined frs 226–255 (700 mg), and applied on a a column of silica gel (35 g), and eluted with mixtures of petrol-CHCl_3_, wherein 250 mL sfrs were collected as follows: Sfrs 1–77 (petrol-CHCl_3_, 1:1), 78–142 (petrol-CHCl_3_, 3:7), 143–220 (petrol-CHCl_3_, 1:9), 221–255 (CHCl_3_). Sfrs 51–63 were combined (50 mg) and crystalized in a mixture of CHCl_3_ and petrol to give 26 mg of byssochlamic acid. Sfrs 125–220 were combined (160 mg) and crystalized in a mixture of CHCl_3_ and petrol to give 120 mg of cyathisterone [10]. Frs 361–420 were combined (312 mg) and purified by TLC (Silica gel G_254_, petrol-CHCl_3_-EtOAc-HCO_2_H, 1:8:1:0.01) to give 9 mg of byssochlamic acid and 20.3 mg of dankasterone A (**2**) [11]. The combined frs 256–360 (1.33 g) and 421–443 (4.9 g) were joined together and applied on a column of silica gel (65 g), and eluted with mixtures of petrol-CHCl_3_ and CHCl_3_-Me_2_CO, wherein 250 mL sfrs were collected as follows: Sfrs 1–250 (petrol-CHCl_3_, 1:1), 251–386 (petrol-CHCl_3_, 3:7), 387–605 (petrol-CHCl_3_, 1:9), 606–858 (CHCl_3_), 859–915 (CHCl_3_-Me_2_CO, 9:1). Sfrs 316–365 were combined (35 mg) and purified by TLC (Silica gel G_254_, petrol-CHCl_3_-EtOAc-HCO_2_H, 1:8:1:0.01) to give 10.5 mg of chevalone B [12] and 4 mg of dankasterone A (**2**). Sfrs 418–480 were combined (11.3 mg) and crystallized in MeOH to give 7 mg of aszonalenin [13] Fr 449 (736 mg) was crystallized in MeOH to give 138 mg of secalonic acid A [14]. Frs 450–452 were combined (1.7 g) and applied on a column of silica gel (100 g), and eluted with mixtures of petrol-CHCl_3_ andCHCl_3_-Me_2_CO, wherein 250 mL sfrs were collected as follows: Sfrs 1–23 (petrol-CHCl_3_, 1:1), 24–58 (petrol-CHCl_3_, 3:7), 59–150 (petrol-CHCl_3_, 1:9), 151–594 (CHCl_3_), 595–649 (CHCl_3_-Me_2_CO, 19:1), 650–735 (CHCl_3_-Me_2_CO, 9:1), 736–955 (CHCl_3_-Me_2_CO, 9:1). Sfrs 601–602 were combined and crystalized in MeOH to give 10.5 mg of paecilin E (**1**). Frs 453–457 were combined (1.49 g) and crystalized in MeOH to give 118 mg of secalonic acid A. The mother liquor was applied on a column of Sephadex LH-20 (10 g) and eluted with a 1:1 mixture of MeOH-CH_2_Cl_2_, wherein 20 sfrs of 10 mL were collected. Sfrs 10–12 were combined (10.6 mg) and crystalized in MeOH to give another 8.7 mg of helvoloic acid. Frs 617–623 were combined (39 mg) and applied on a column of Sephadex LH-20 (10 g) and eluted with a 1:1 mixture of MeOH: CH_2_Cl_2_, wherein 30 sfrs of 3 mL were collected. Sfrs 17–30 were combined and crystalized in MeOH to give 4.5 mg of fellutanine A [13]. Frs 631–675 were combined (3.61 g) and crystallized in MeOH to give further 68.3 mg of secalonic acid A.

The crude ethyl acetate extract of *N. tsunodae* KUFC 9213 was dissolved in 500 mL of CHCl_3_, and then washed with H_2_O (3 × 500 mL). The organic layers were combined and dried with anhydrous Na_2_SO_4_, filtered and evaporated under reduced pressure to give 60 g of the crude chloroform extract, which was applied on a column of silica gel (410 g), and eluted with mixtures of petrol-CHCl_3_, CHCl_3_-Me_2_CO and CHCl_3_-MeOH , wherein 250 mL fractions were collected as follows: Frs 1–99 (petrol-CHCl_3_, 1:1), 100–201 (petrol-CHCl_3_, 3:7), 202–219 (petrol-CHCl_3_, 1:9), 220–349 (CHCl_3_-Me_2_CO, 9:1), 350–391 (CHCl_3_-Me_2_CO, 7:3), 392–437 (CHCl_3_-MeOH, 9:1), 438–455 (CHCl_3_-MeOH, 7:3) and 456–459 (MeOH). Frs 134–196 were combined (2.0 g) and purified by TLC (Silica gel G_254_, CHCl_3_-petrol-HCO_2_H, 14:5:1) to give 40.5 mg of byssochlamic acid [7]. Frs 226–234 were combined (4.0 g) and applied on a column of silica gel (33 g), and eluted with mixtures of petrol-CHCl_3_, CHCl_3_, and CHCl_3_-Me_2_CO, wherein 100 mL subfractions (sfrs) were collected as follows: Sfrs 1–5 (petrol-CHCl_3_, 7:3), 6–18 (petrol-CHCl_3_, 3:2), 19–20 (petrol-CHCl_3_, 1:1), 21–34 (petrol-CHCl_3_, 3:7), 25–30 (petrol-CHCl_3_, 9:1), 31–42 (CHCl_3_) and 43–48 (CHCl_3_-Me_2_CO, 9:1). Sfrs 24–30 were combined (211 mg) and crystallized in MeOH to give 64 mg of byssochlamic acid and 35 mg of hopan-3β,22 diol [15]. Sfrs 31–42 were combined (174 mg) and crystallized in MeOH to give further 23.4 mg of byssochlamic acid. Frs 235–244 were combined (1.75 g) and applied on a column of silica gel (45 g), and eluted with mixtures of petrol-CHCl_3_ and CHCl_3_, wherein 100 mL sfrs were collected as follows: Sfrs 1–9 (petrol-CHCl_3_, 7:3), 20–32 (petrol-CHCl_3_, 3:2), 33–45 (petrol-CHCl_3_, 1:1), 46–60 (petrol-CHCl_3_, 3:7), 61–112 (petrol-CHCl_3_, 1:9) and 113–115 (CHCl_3_). Sfrs 1–5 were combined and purified by TLC (Silica gel G_254_, CHCl_3_-Me_2_CO-HCO_2_H, 97:3:0.1) to give 4.6 mg of byssochlamic acid and 12.4 mg of chevalone C [16]. Sfrs 6–75 were combined (91 mg) and crystalized in MeOH to give further 15 mg of byssochlamic acid. Sfrs 76–114 were combined (863 mg) and purified by TLC (Silica gel G_254_, CHCl_3_-Me_2_CO-HCO_2_H, 97:3:0.1) to give an additional 15.7 mg of byssochlamic acid, 22.4 mg of chevalone C and 39.3 mg of sartorypyrone B [5]. Frs 245–263 were combined (1.53 g) and applied on a column of silica gel (45 g), and eluted with mixtures of petrol-CHCl_3_, CHCl_3_, CHCl_3_-Me_2_CO, and Me_2_CO, wherein 100 mL sfrs were collected as follows: Sfrs 1–12 (petrol-CHCl_3_, 7:3), 13–20 (petrol-CHCl_3_, 3:2), 21–40 (petrol-CHCl_3_, 1:1), 41–50 (petrol-CHCl_3_, 2:3), 51–68 (petrol-CHCl_3_, 3:7), 69–85 (petrol-CHCl_3_, 1:4), 86–100 (petrol-CHCl_3_, 1:9), 101–122 (CHCl_3_), 123–148 (CHCl_3_-Me_2_CO, 9:1), 149–158 (Me_2_CO). Sfrs 23–123 were combined (57 mg) and crystalized in MeOH to give 12 mg of byssochlamic acid and 7.1 mg of sartorypyrone B. Frs 264–312 were combined (1.12 g) and applied on a column of silica gel (18 g), and eluted with mixtures of petrol-CHCl_3_ and CHCl_3_, wherein 100 mL sfrs were collected as follows: Sfrs 1–17 (petrol-CHCl_3_, 7:3), 18–48 (petrol-CHCl_3_, 3:2), 49–72 (petrol-CHCl_3_, 1:1), 73–76 (petrol-CHCl_3_, 2:3), 77–90 (petrol-CHCl_3_, 3:7), 91–100 (petrol-CHCl_3_, 1:9), 116 (CHCl_3_). Sfrs 16–68 were combined (93 mg) and crystalized in MeOH to give 33 mg of byssochlamic acid. Sfrs 69–115 were combined (711 mg) and purified by TLC (Silica gel G_254_, CHCl_3_-Me_2_CO-HCO_2_H, 4:1:0.01) to give to 14.1 mg of lumichrome [21] and 8.0 mg of helvolic acid [5]. Frs. 313–352 were combined (487 mg) and applied on a Sephadex LH-20 column (10 g) and eluted with MeOH, wherein 20 mL of 42 sfrs were collected. Sfrs 15–42 were combined (104 mg) and purified by TLC (Silica gel G_254_, CHCl_3_-Me_2_CO-HCO_2_H, 4:1:0.01) to give 10 mg of byssochlamic acid, 7.8 mg of helvolic acid, 4.7 mg of lumichrome, 10.6 mg of (3β,5α,22*E*)-3,5-dihydroxyergosta-7,22-dien-6-one (**4**) [28] and 21.6 mg of chromanol (**3**). Fractions 400–420 were combined (1.47 g) and applied on a Sephadex LH-20 column (20 g) and eluted with MeOH, wherein 20 mL of 42 sfrs were collected. Sfr 23–42 were combined (306 mg) and purified by TLC (Silica gel G_254_, CHCl_3_-Me_2_CO-HCO_2_H, 9:1:0.01) to give to 25.4 mg of byssochlamic acid and 5.3 mg of harmane [20]. Frs 421–440 were combined (1.33 g) and applied on a Sephadex LH-20 column (20 g) and eluted with MeOH, wherein 20 mL of 33 sfrs were collected. Sfrs 18–33 were combined (126 mg) and crystalized in MeOH to give additional 42.2 mg of harmane.

#### 3.3.1. Paecilin E (**1**)

White crystal; mp 203–204 °C. ${\left[\alpha \right]}_{D}^{20}$ +154 (*c* 0.03, MeOH); IR (KBr) υ_max_ 3444, 2959, 2920, 1790, 1738, 1645, 1470, 1261cm^−1^. For ^1^H and ^13^C spectroscopic data (DMSO, 500 and 125 MHz), see Table 2; (+)-HRESIMS *m*/*z* 639.1718 (M + H)^+^ (calcd. for C_32_H_31_O_14_, 639.1714).

#### 3.3.2. Dankasterone (**2**)

White crystal; mp 135–137 °C. ${\left[\alpha \right]}_{D}^{20}$ +166 (*c* 0.04, CHCl_3_); IR (KBr) υ_max_ 2959, 2924, 1727, 1710, 1536, 1462 cm^−1^. For ^1^H and ^13^C spectroscopic data (CDCl_3_, 500.13 and 125.8 MHz), see Table S1; (+)-HRESIMS *m*/*z* 347.1111 (M + Na)^+^ (calcd. for C_16_H_20_O_7_ Na, 341.1107). (+)-HRESIMS *m*/*z* 425.3054 (M + H)^+^ (calcd. for C_28_H_41_O_3_, 425.3056).

#### 3.3.3. (1*R*, 8*S*, 9*R*)-1,9-Dihydroxy-8-(2-hydroxypropan-2-yl)-4-methoxy-5-methyl-1,7,8,9-tetrahydro-3*H*-furo[3,4-f]chromen-3-one (**3**)

White crystal; mp 223–224 °C. ${\left[\alpha \right]}_{D}^{20}$ –80 (*c* 0.05, CHCl_3_); IR (KBr) υ_max_ 3467, 3434, 3018, 2969, 1743, 1597, 1507, 1262 cm^−1^. For ^1^H and ^13^C spectroscopic data (DMSO, 300.13 and 75.4 MHz), see Table 2; (+)-HRESIMS *m*/*z* 347.1111 (M + Na)^+^ (calcd. for C_16_H_20_O_7_ Na, 341.1107). 

#### 3.3.4. (3β,5α,22*E*)-3,5-Dihydroxyergosta-7,22-dien-6-one (**4**)

White amorphous solid; ${\left[\alpha \right]}_{D}^{20}$ +60 (*c* 0.05, CHCl_3_); For ^1^H and ^13^C spectroscopic data (CDCl_3_, 500.13 and 125.8 MHz), see Table S2. (+)-HRESIMS *m*/*z* 429.3388 (M + H)^+^ (calcd. for C_28_H_45_O_3_, 429.3369).

### 3.4. Electronic Circular Dichroism (ECD)

The ECD spectrum of **4** (1.6 mM in methanol) was obtained in a Jasco J-815 CD spectropolarimeter with a 0.01 mm cuvette and eight accumulations. Dihedral driver and MMFF95 minimizations were done in Chem3D Ultra (Perkin-Elmer Inc., Waltham, MA, USA). All DFT minimizations and ECD spectral calculations (TD-DFT) were performed with Gaussian 09W (Gaussian Inc., Wallingford, CT, USA) using the APFD/6-311+G (2d, p) method/basis set [32] with IEFPCM solvation model of methanol. The simulated spectral lines (Figure 4) were obtained by summation of Gaussian curves, as recommended in Stephens and Harada [33]. A line broadening of 0.4 eV was applied to all transitions to generate the calculated spectral lines.

### 3.5. X-ray Crystal Structure of ***1*** and ***3***

Diffraction data were collected with a Gemini PX Ultra equipped with CuK_α_ radiation (λ = 1.54184 Å). The structures were solved by direct methods using SHELXS-97 and refined with SHELXL-97 [34]. Carbon, oxygen and sulfur atoms were refined anisotropically. Hydrogen atoms were either placed at their idealized positions using appropriate HFIX instructions in SHELXL, and included in subsequent refinement cycles, or were directly found from difference Fourier maps and were refined freely with isotropic displacement parameters. Full details of the data collection and refinement and tables of atomic coordinates, bond lengths and angles, and torsion angles have been deposited with the Cambridge Crystallographic Data Centre.

Paecilin E (**1**). Crystals were monoclinic, space group P2_1_, cell volume 1487.9(2) Å^3^ and unit cell dimensions *a* = 13.5112(7) Å, *b* = 8.1824(11) Å and *c* = 14.7531(9) Å and β = 114.179(7)° (uncertainties in parentheses). The refinement converged to *R* (all data) = 5.27% and *wR*_2_ (all data) = 10.31%. The absolute structure was established with confidence (flack *x* parameter 0.0(2)). Diffraction data were collected at 148 K. CCDC 1579859.

*(1R, 8S, 9R)-1,9-Dihydroxy-8-(2-hydroxypropan-2-yl)-4-methoxy-5-methyl-1,7,8,9-tetrahydro-3H-furo[3,4-f]chromen-3-one* (**3**). Crystals were triclinic, space group P1, cell volume 773.78(18) Å^3^ and unit cell dimensions *a* = 9.1295(12) Å, *b* = 9.2537(14) Å and *c* = 10.4317(12) Å and angles α = 94.622(11)°, β = 104.310(11)° and γ = 112.486(13)° (uncertainties in parentheses). The refinement converged to *R* (all data) = 14.12% and *wR*_2_ (all data) = 29.88%. Diffraction data were collected at 291 K. CCDC 1579876.

### 3.6. Antibacterial Activity Bioassays

#### 3.6.1. Bacterial Strains and Growth Conditions

For reference, a clinical isolate sensitive to the most commonly used antibiotic families, and four multidrug-resistant bacterial strains were used in this study. The Gram-positive bacteria comprised *Staphylococcus aureus* ATCC 29213, *Enterococcus faecalis* ATCC 29212, a clinical isolate *S. aureus* 40/61/24, MRSA *S. aureus* 66/1 isolated from public buses [35], and VRE *E. faecalis* A5/102 and VRE *E. faecalis* B3/101 isolated from river water [36]. The Gram-negative bacteria used were *Escherichia coli* ATCC 25922, *Pseudomonas aeruginosa* ATCC 27853, and a clinical isolate ESBL *E. coli* SA/2. Frozen stocks of all strains were grown in Mueller-Hinton agar (MH-BioKar diagnostics, Allone, France) at 37 °C. All bacterial strains were sub-cultured in MH agar and incubated overnight at 37 °C before each assay.

#### 3.6.2. Antimicrobial Susceptibility Testing

The minimum inhibitory concentration (MIC), which was used for determining the antibacterial activity of each compound, was determined according to the method described previously by May Zin et al. [37].

#### 3.6.3. Biofilm Formation Inhibition Assay

The effect of the compounds on biofilm formation was assessed using crystal violet staining as previously described by May Zin et al. [37].

#### 3.6.4. Antibiotic Synergy Testing

Evaluation of the combined effect of the compounds and clinical relevant antimicrobial drugs was performed according to the method previously described by May Zin et al. [37].

## 4. Conclusions

Chemical investigation of the culture of the marine-derived fungus *Neosartorya fennelliae* KUFA 0811, isolated from the marine sponge *Clathria reinwardtii*, resulted in the isolation of the previously undescribed 6-8 dimer of substituted 3,5-dihydrochromone which we have named paecilin E (**1**), and the previously reported metabolites including β-sitostenone, ergosta-4,6,8 (14), 22-tetraen-3-one, cyathisterone, byssochlamic acid, dehydromevalonic acid lactone, chevalone B, aszonalenin, dankasterone A (**2**), helvolic acid, secalonic acid A and fellutanine A. Re-examination of the culture of *N. tsunodae* KUFC 9213, led to the isolation of the chromanol derivative (**3**), in addition to sartorypyrone B and helvolic which were previously isolated from this fungus, and other known compounds including byssochlamic acid, hopan-3β,22-diol (**5**), chevalone C, (3β,5α,22*E*)-3,5-dihydroxyergosta-7,22-dien-6-one (**4**), the alkaloid harmane (**7**) and lumichrome (**6**). The absolute configurations of the stereogenic carbons of the previously undescribed paecilin E (**1**) and the chromanol derivative (**3**) were unambiguously established by X-ray analysis. Although (3β,5α,22*E*)-3,5-dihydroxyergosta-7,22-dien-6-one (**4**) has been reported from several sources, the absolute configuration of its C-5 had never been determined unambiguously by any modern techniques. By comparison of the experimental and calculated ECD spectra, we determined conclusively the absolute configuration of C-5 as 5*R*. Paecilin E (**1**), dankasterone A (**2**), the chromanol derivative (**3**) and some of the isolated compounds which have not been previously tested for antibacterial activity, i.e., (3β,5α,22*E*)-3,5-dihydroxyergosta-7,22-dien-6-one (**4**), hopan-3β,22-diol (**5**), lumichrome (**6**) and harmane (**7**) were tested for their antibacterial activity against Gram-positive and Gram-negative bacteria of four reference strains, a clinical isolate sensitive to the most commonly used antibiotic families, and four multidrug-resistant isolates from the environment. Only paecilin E (**1**) and dankasterone A (**2**) were able to inhibit growth of Gram-positive bacteria. While paecilin E (**1**) exhibited an inhibitory effect on both *S. aureus* ATCC 29213 and *E. faecalis* ATCC 29212 with MIC values of 32 μg/mL and 16 μg/mL, respectively, dankasterone (**2**) was only effective against *E. faecalis* ATCC 29212 and VRE *E. faecalis* A5/102, with MIC of 32 μg/mL and 64 μg/mL, respectively. Despite a great structural diversity of the secondary metabolites produced by these two marine-derived species of *Neosartorya*, a majority of them did not possess the antibacterial activity. Nevertheless, it does not mean that they do not have other interesting biological activities. Therefore, more biological assays will be performed in the future.

## Figures and Tables

**Table 1:** ^1^H and ^13^C Nuclear magnetic resonance (NMR) (DMSO, 500 and 125 MHz) and Heteronuclear Multiple Bond Correlation (HMBC) assignment for **1**.

Position	δ_C_, Type	δ_H_, (*J* in Hz)	COSY	HMBC
2	85.4, C	-		
3α	32.9, CH_2_	3.58, d (17.4)	H-3β	C-2, 4, 9, 10
β		3.05, d (17.4)	H-3α	C-4, 4a
4	194.9, CO	-		
5	107.5, C	-		
6	109.2, CH	6.61, d (8.6)	H-7	C-4a, 8
7	140.9, CH	7.50, d (8.6)	H-6	C-5, 8a
8	114.6, C	-		
9	168.9, CO (Ac)	-		
OMe-9	53.3, CH_3_	3.70, s		C-9
10	81.7, CH	4.85, d (7.3)	H-11	C-2, 3, 12, 13, 14
11	32.9, CH	2.85, m	H-10, H_2_-12, Me-14	C-2, 13, 14
12α	35.3, CH_2_	1.75, dd (17.0, 9.9)	H-11, 12β	C-10, 13, 14
β		2.41, dd (17.0, 8.4)	H-11, 12 α	C-10, 13, 14
13	174.9, CO	-		
14	14.1, CH_3_	1.06, d (7.1)	H-11	C-10, 11, 12
2′	83.9, C	-		
3′α	39.2, CH_2_	3.57, d (17.4)	H-3′β	C-2′, 4′, 9′, 10′
β		3.09, d (17.4)	H-3′α	C-4′, 4′a
4′	195.3, CO	-		
4′a	107.0, C	-		
5′	158.1, C	-		
6′	116.6, C	-		
7′	140.6, CH	7.61, d (8.6)	H-8′	C-5′, 8′a, 8
8′	107.4, CH	6.60, d (8.6)	H-7′	C-6′, 8′a
8′a	158.2, C	-		
9′	169.3, CO (Ac)	-		
OMe-9′	53.3, CH_3_	3.69, s		C-9′
10′	81.9, CH	4.97, d (6.7)	H-11′	C-3′, 11′, 13′, 14′
11′	32.5, CH	2.97, m	H-10′, 11′, 12′a, 12′β	C-2′, 13′, 14′
12′α	36.9, CH_2_	2.33, dd (17.0, 5.4)	H-11′, 12′β	C-10′, 13′, 14′
β		2.86, dd (17.0, 8.1)	H-11′, 12′α	C-10′, 13′, 14′
13′	175.5, CO	-		
14′	14.6, CH_3_	1.17, d (7.1)	H-11′	C-10′, 11′, 12′
OH-5	-	11.56, s		C-4a, 5, 6
OH-5′	-	11.83, s		C-4′a, 5′, 6′

**Table 2:** ^1^H and ^13^C NMR (CDCl_3_, 300 MHz and 75 MHz) and HMBC assignment for **3**.

Position	δ_C_, Type	δ_H_, (*J* in Hz)	COSY	HMBC	NOESY
1	95.6, CH	6.64, s	-	C-3	OH-1, H-9
3	166.1, CO	-			
3a	109.4, C	-			
4	155.9, C	-			
5	120.0, C	-			
5a	158.4, C				
7α	63.9, CH_2_	4.29, dd (12.0, 10.6)	H-7β, 8	C-5a, 8, 9	H-7β
β		4.53, dd (11.6, 2.4)	H-7α	C-5a, 8, 9	
8	46.6, CH	1.79, dt (11.9, 2.8)	H-7α	C-2′, 7	H-8, Me-1′, 3′
9	57.8, CH	5.16, br	-		
9a	146.8, C	-			
9b	117.4, C	-			
10	8.6, CH_3_	2.05, s	-	C-3a, 4, 5, 5a, 9a	OMe-4
1′	28.4, CH_3_	1.27, s	-	C-2′, 3′, 8	H-8, OH-2′, Me-3′
2′	69.9, C	-			
3′	27.7, CH_3_	1.24, s		C-1′, 2′, 8	H-8, OH-2′, Me-1′

**Table 3:** Antibacterial activity of paecilin E (**1**) and dankasterone A (**2**). MIC and MBC are expressed in μg/mL.

	Paecilin E (1)		Dankasterone A (2)	
Bacterial strain	MIC	MBC	MIC	MBC
*E.coli* ATCC 25922	>64	>64	>64	>64
*E.coli* SA/2 (ESBL)	>64	>64	>64	>64
*P. aeruginosa* ATCC 27853	>64	>64	>64	>64
*E. faecalis* ATCC29212	16	>64	32	>64
*E. faecalis* A5/102 (VRE)	64	>64	64	>64
*E. faecalis* B3/101 (VRE)	>64	>64	>64	>64
*S. aureus* ATCC 29213	32	>64	>64	>64
*S. aureus* 40/61/24	>64	>64	>64	>64
*S. aureus* 66/1 (MRSA)	>64	>64	>64	>64

MIC = mininmum inhibitory concentration; MBC = minimum batericidal concentration.

## Supplementary Materials

The following are available online at www.mdpi.com/1660-3397/15/12/375/s1, Figure S1: Structures of metabolites isolated from *Neosartorya tsunodae* KUFC 9231 and *N. fennelliae* KUFA 0811, Figures S2–S48: 1D and 2D NMR spectra of isolated compounds, Figure S49: Ortep view of dankasterone A (**2**), Table S1: ^1^H and ^13^C NMR (CDCl_3_, 500 MHz and 125 MHz) and HMBC assignment for **2,** Table S2: ^1^H and ^13^C NMR (CDCl_3_, 500 MHz and 125 MHz) of **4**.
