# Supplementary material for: A New Dihydrochromone Dimer and Other Secondary Metabolites from Cultures of the Marine Sponge-Associated Fungi Neosartorya fennelliae KUFA 0811 and Neosartorya tsunodae KUFC 9213
Source: Mar Drugs. 2017 Dec 1;15(12):375. doi: 10.3390/md15120375 (PMC5742835; doi:10.3390/md15120375)
Supplement: Supplementary file 1 [file marinedrugs-15-00375-s001.pdf]

Supplementary Materials

A New Dihydrochromone Dimer and Other Secondary Metabolites from Cultures of the Marine Sponge-Associated Fungi *Neosartorya fennelliae* KUFA 0811 and *Neosartorya tsunodae* KUFC 9213

Decha Kumla [a, b†], Tin Shine Aung [a, b†], Suradet Buttachon [a, b], Tida Dethoup [c], Luís Gales [a, d], José A. Pereira [a, b], Ângela Inácio [b], Paulo M. Costa [a, b], Michael Lee [e], Nazim Sekeroglu [f], Artur M. S. Silva [g], Madalena M. M. Pinto [b, h], Anake Kijjoa [a, b*]

Comentado [M1]: Changed § to this symbol, please confirm. **AK**: It is OK.

Comentado [M2]: Changed § to this symbol, please confirm. **AK**: It is OK.

[a] *ICBAS-Instituto de Ciências Biomédicas Abel Salazar, Rua de Jorge Viterbo Ferreira, 228, 4050-313 Porto, Portugal. E-mail: decha1987@hotmail.com (DK), tinshineaung@gmail.com (TSA), jpereira@icbas.up.pt (JAP), pmcosta@icbas.up.pt (PMC).*

[b] *Interdisciplinary Centre of Marine and Environmental Research (CIIMAR), Terminal de Cruzeiros do Porto de Lexões, Av. General Norton de Matos s/n, 4450-208, Matosinhos, Portugal. E-mail: nokrari_209@hotmail.com (SB), angelainacio@gmail.com (AI).*

[c] *Department of Plant Pathology, Faculty of Agriculture, Kasetsart University, Bangkok 10240, Thailand. E-mail: tdethoup@yahoo.com.*

[d] *Instituto de Biologia Molecular e Celular (i3S-IBMC), Universidade do Porto, Rua de Jorge Viterbo Ferreira, 228, 4050-313 Porto, Portugal. E-mail: lgales@ibmc.up.pt.*

[e] *Department of Chemistry, University of Leicester, University Road, Leicester LE 7 RH, UK. E-mail: ml34@leicester.ac.uk.*

[f] *Medicinal and Aromatic Plant Programme, Plant and Animal Sciences Department, Vocational School, Kilis 7 Aralık University, 79000, Kilis, Turkey. E-mail: nsekeroglu@gmail.com.*

[g] *Departamento de Química & QOPNA, Universidade de Aveiro, 3810-193 Aveiro, Portugal. E-mail: artur.silva@ua.pt.*

[h] *Laboratório de Química Orgânica, Departamento de Ciências Químicas, Faculdade de Farmácia, Universidade do Porto, Rua de Jorge Viterbo Ferreira, 228, 4050-3 13 Porto, Portugal. E-mail: madalena@ff.up.pt*

†These authors contributed equally to this work.

Comentado [M3]: Changed § to this symbol, please confirm. **AK**: It is OK.

*Corresponding author.

E-mail address: ankijjoa@icbas.up.pt (A. Kijjoa)

Figure S1. Structures of metabolites isolated from *Neosartorya tsunodae* KUFC 9231 and *N. fennelliae* KUFA 0811.

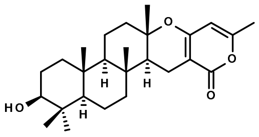

Chevalone B

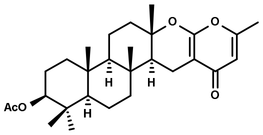

Chevalone C

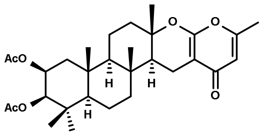

Sartorypyrone B

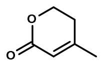

Dehydromevalonic acid lactone

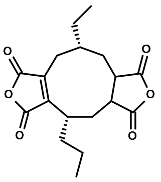

Byssochlamic acid

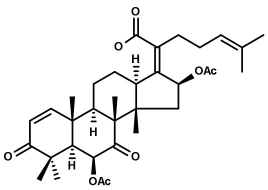

Helvoric acid

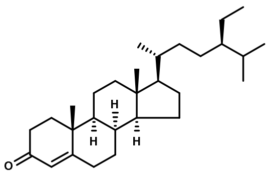

β-Sitostenone

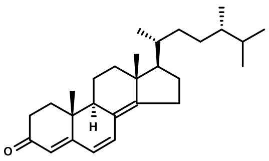

Ergosta-4,6,8(14), 22-tetraen-3-one

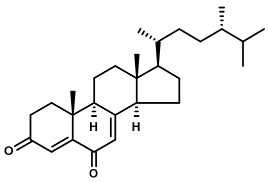

Cyathisterone

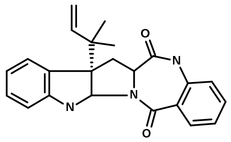

Aszonalenin

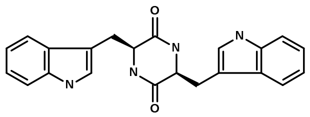

Fellutanine A

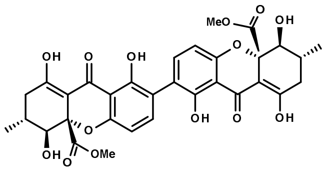

Secalonic acid A

Figure S2. ^{1}H NMR spectrum of byssochlamic acid ($CDCl_3$, 300.13 MHz).

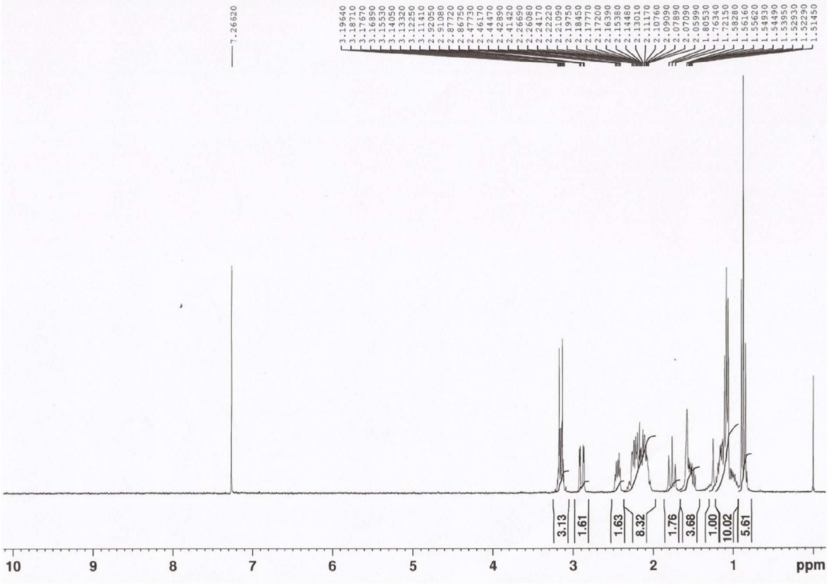

Figure S3. ^{13}C NMR spectrum of byssochlamic acid ($CDCl_3$, 75.4 MHz).

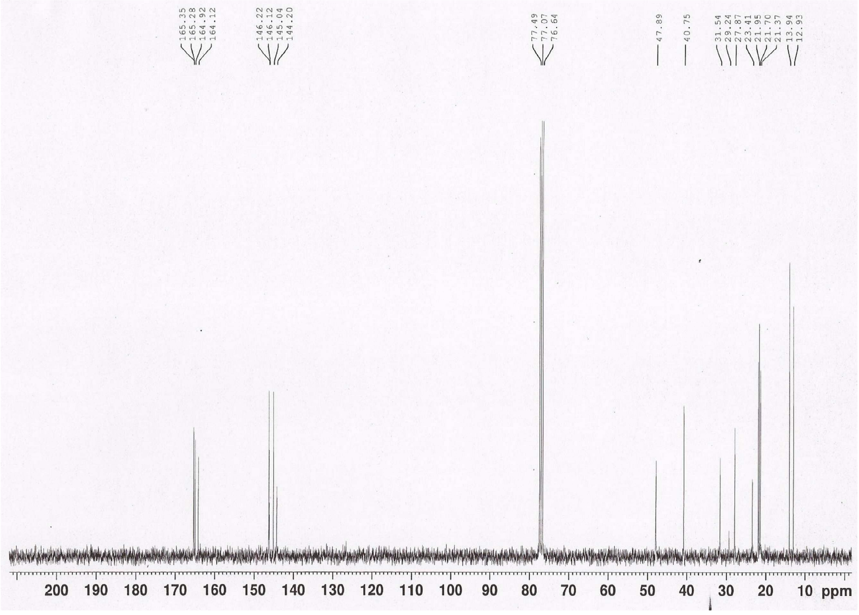

Figure S4. ^{1}H NMR spectrum of hopan-3β, 22-diol ($CDCl_3$, 500.13 MHz).

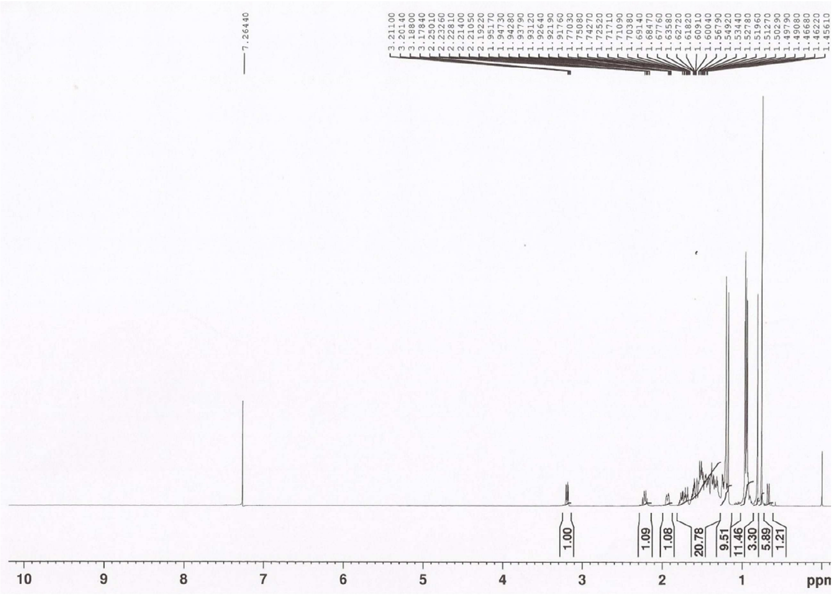

Figure S5. ^{13}C NMR spectrum of hopan-3β, 22-diol ($CDCl_3$, 125.8 MHz).

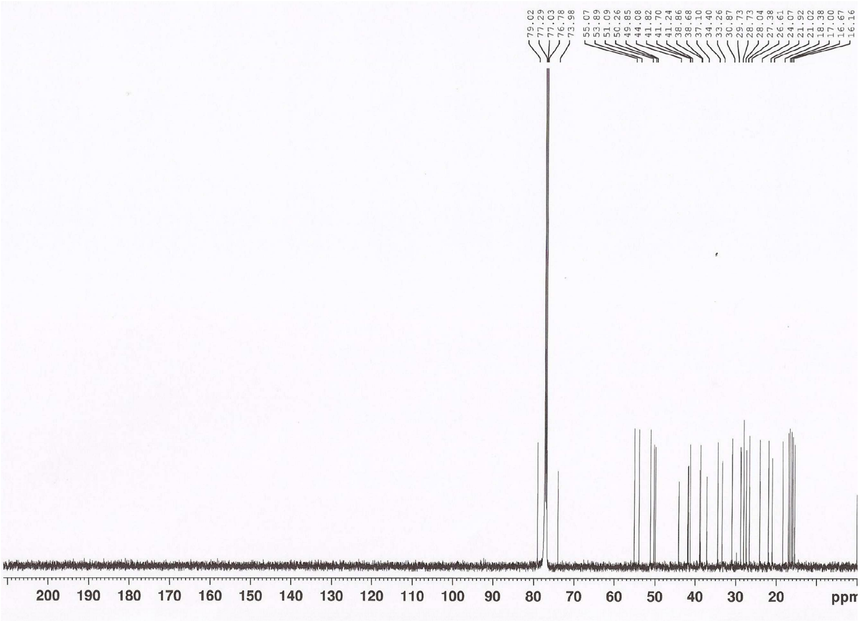

Figure S6. ^{1}H NMR spectrum of chevalone B ($CDCl_3$, 300.13 MHz).

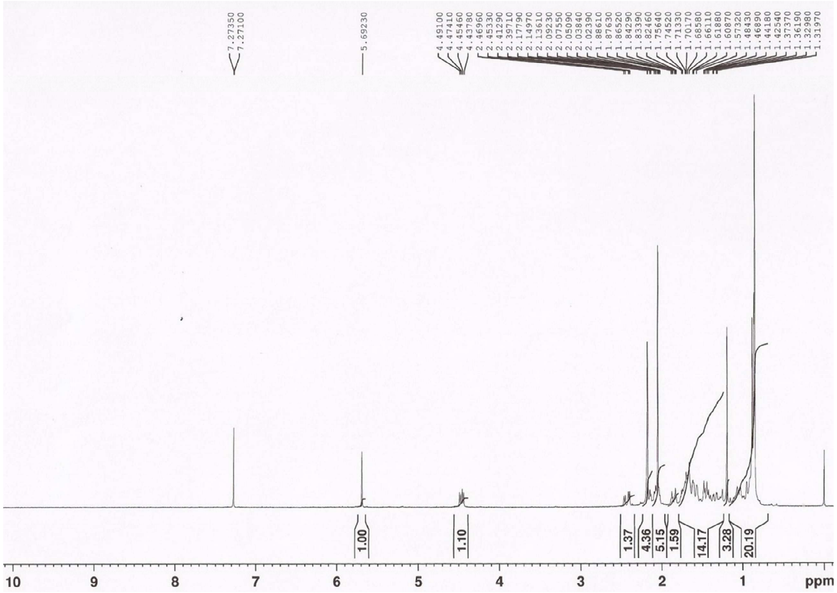

Figure S7. ^{13}C NMR spectrum of chevalone B ($CDCl_3$, 75.4 MHz).

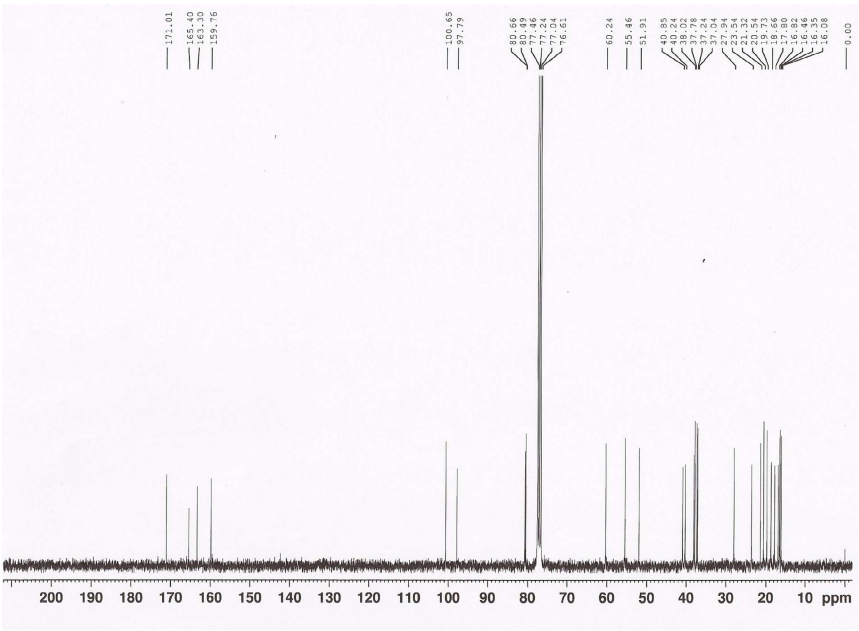

Figure S8. ^{1}H NMR spectrum of chevalone C ($CDCl_3$, 300.13 MHz).

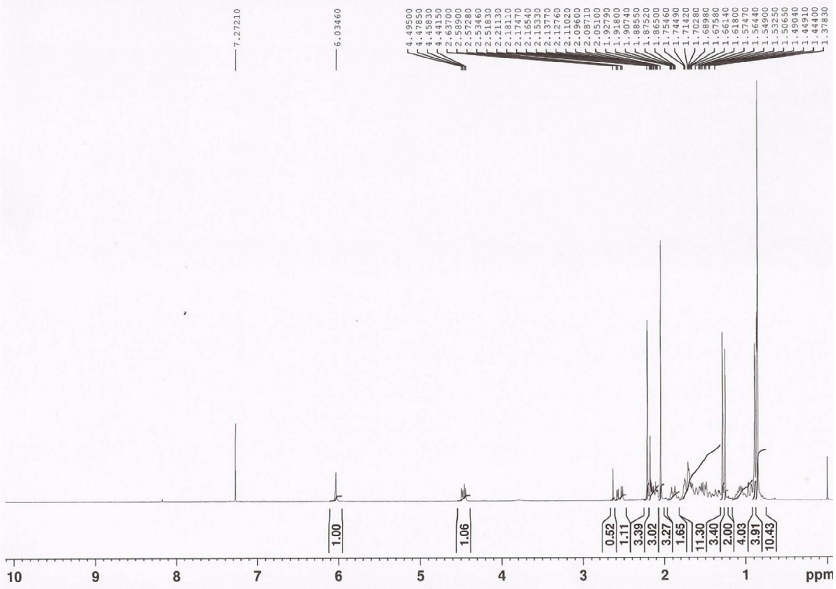

Figure S9. ^{13}C NMR spectrum of chevalone C ($CDCl_3$, 75.4 MHz).

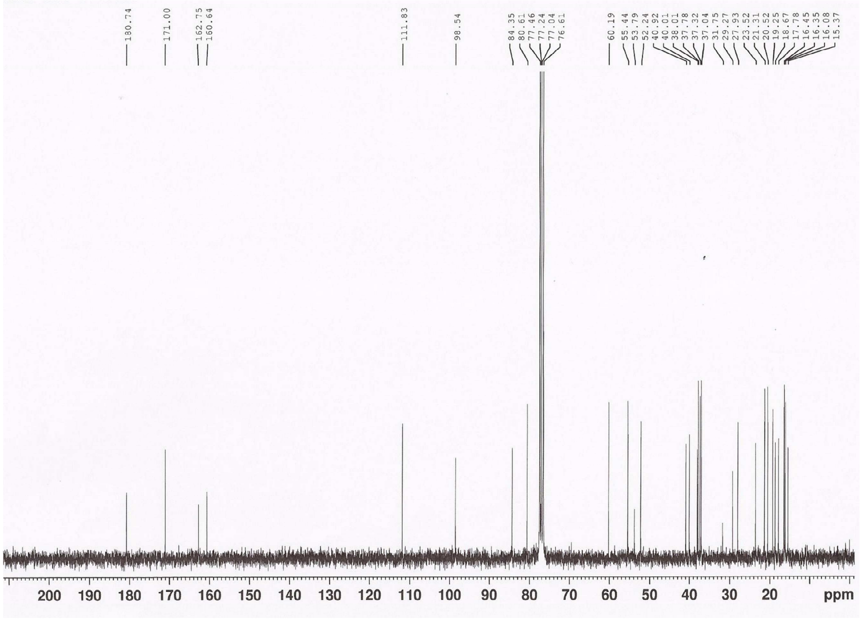

Figure S10. ^{1}H NMR spectrum of sartorypyrone B ($CDCl_3$, 300.13 MHz).

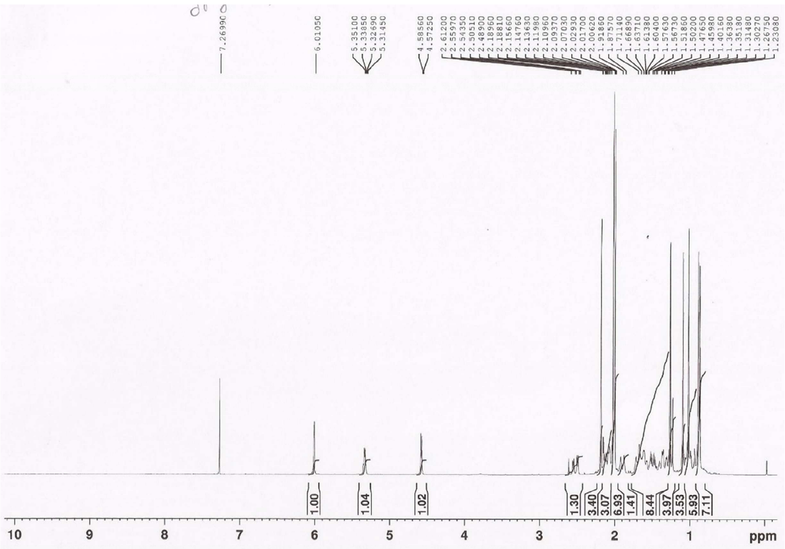

Figure S11. ^{13}C NMR spectrum of sartorypyrone B ($CDCl_3$, 75.4 MHz).

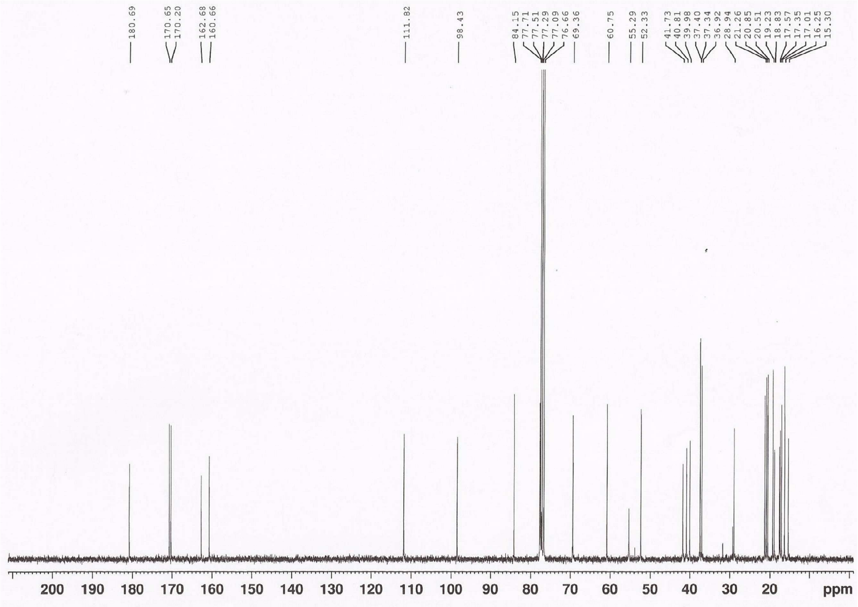

Figure S12. ^{1}H NMR spectrum of helvolic acid ($CDCl_3$, 300.13 MHz).

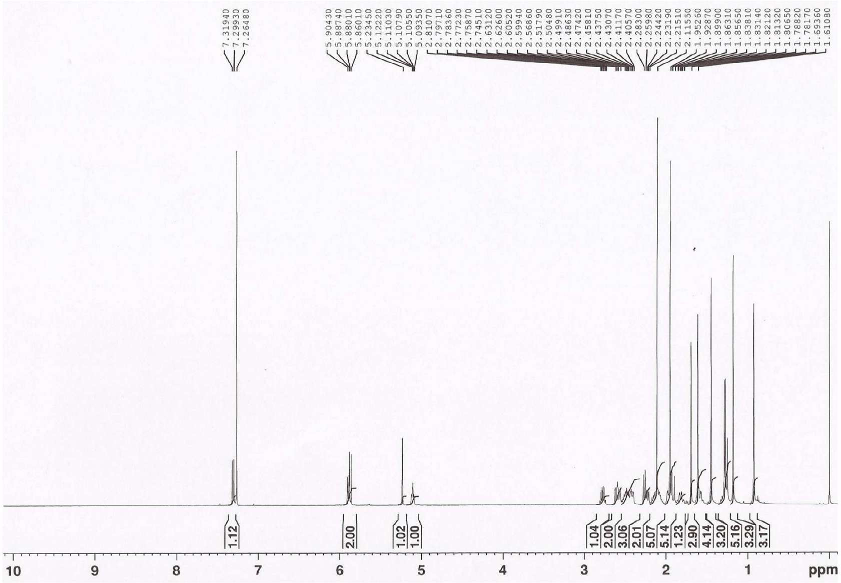

Figure S13. ^{13}C NMR spectrum of helvolic acid ($CDCl_3$, 75.4 MHz).

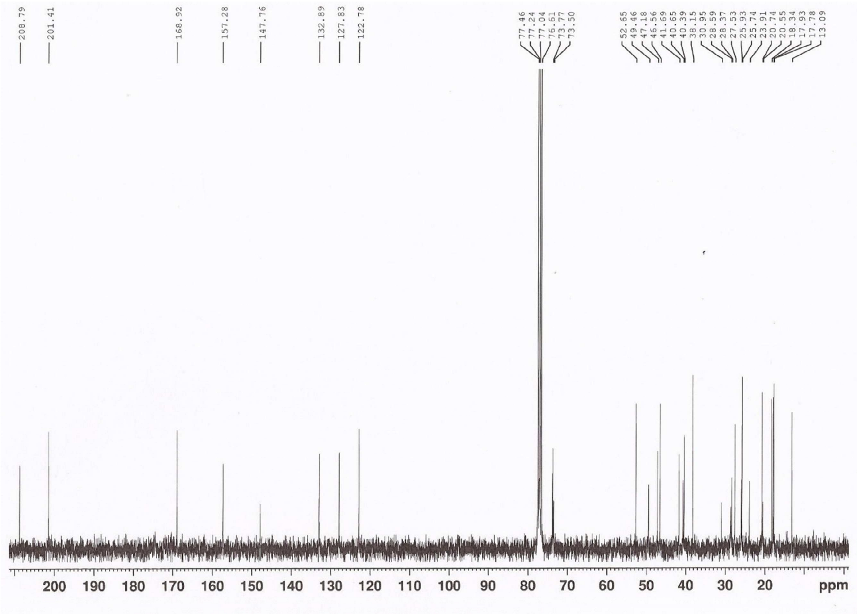

Figure S14. ^{1}H NMR spectrum of lumichrome (DMSO, 300.13 MHz).

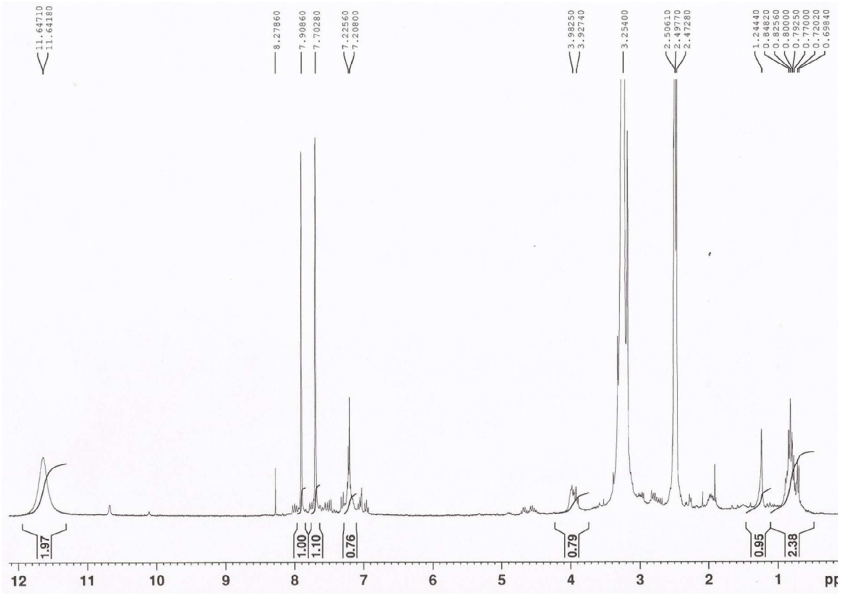

Figure S15. ^{13}C NMR spectrum of lumichrome (DMSO, 75.4 MHz).

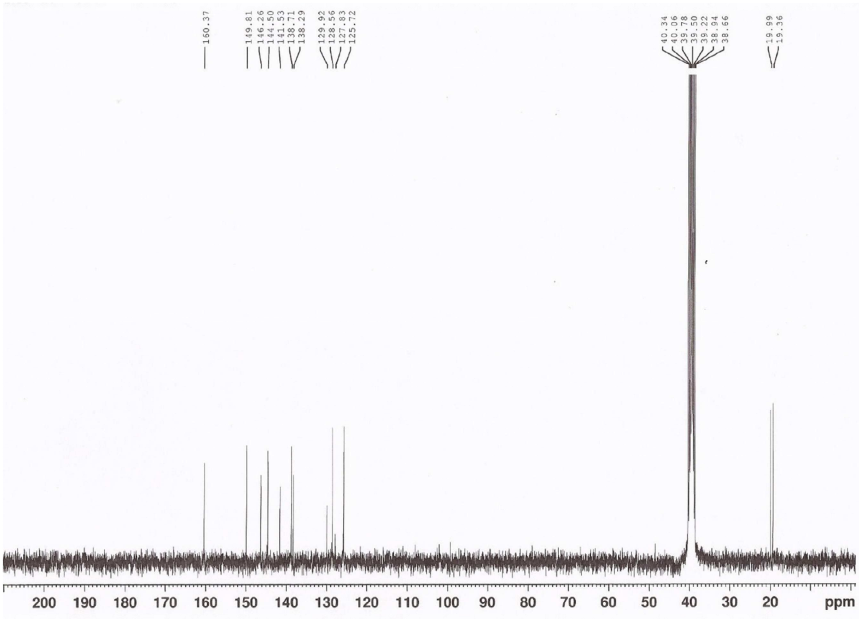

Figure S16. ^{1}H NMR spectrum of harmane (DMSO, 500.13 MHz).

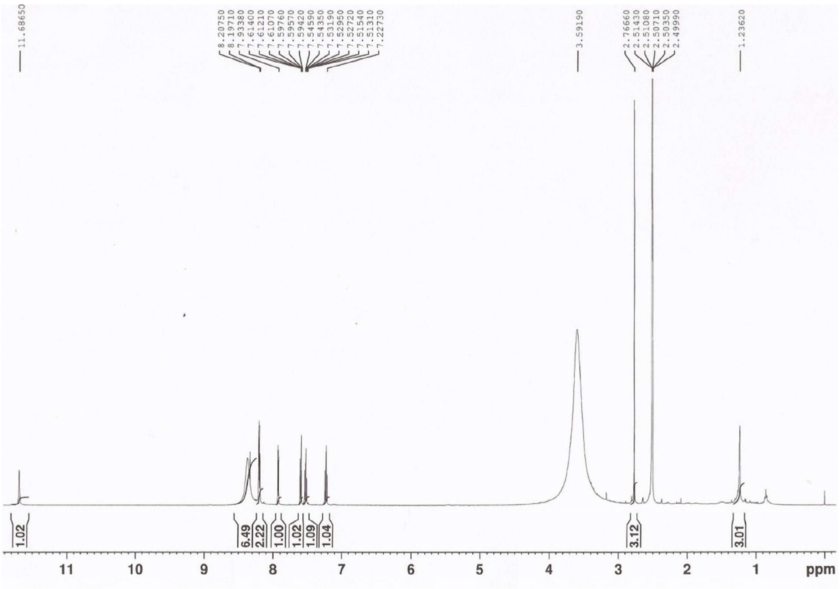

Figure S17. ^{13}C NMR spectrum of harmane (DMSO, 125.8 MHz).

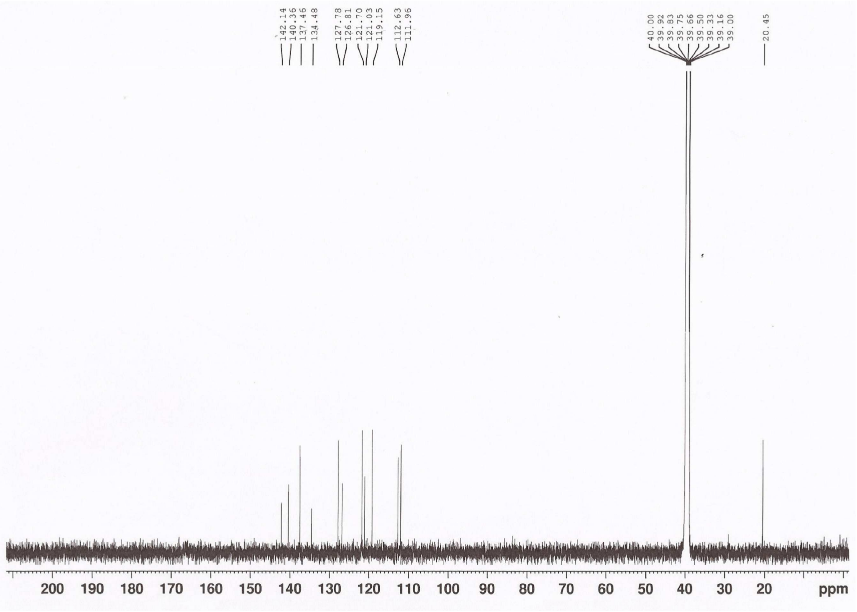

Figure S18. ^{1}H NMR spectrum of β-sitostenone ($CDCl_3$, 300.13 MHz).

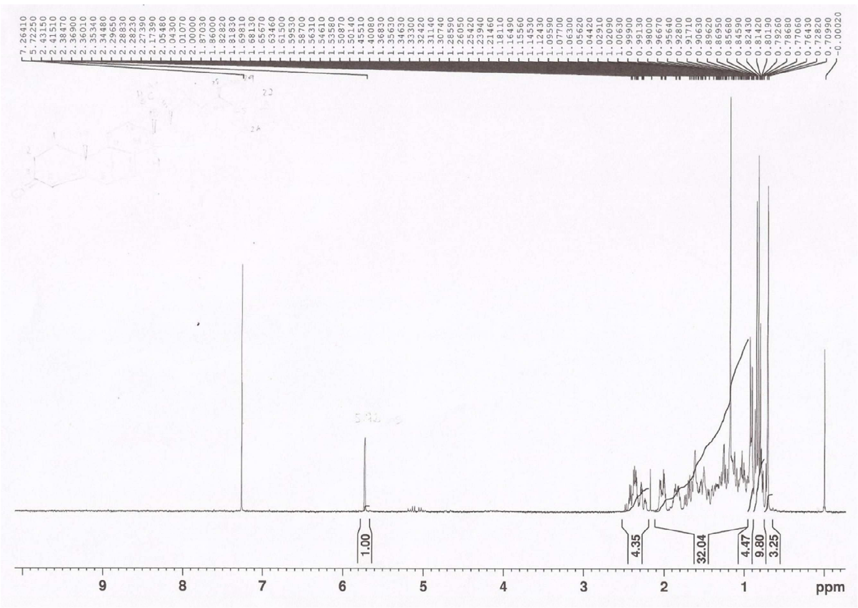

Figure S19. ^{13}C NMR spectrum of β-sitostenone ($CDCl_3$, 75.4 MHz).

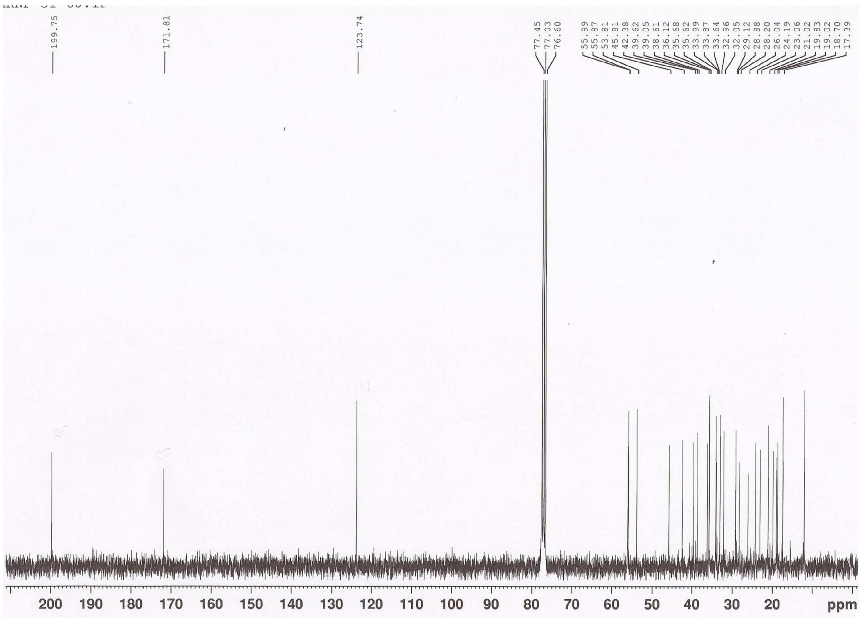

Figure S20. ^{1}H NMR spectrum of ergosta-4,6,8 (14), 22-tetraen-3-one ($CDCl_3$, 300.13 MHz).

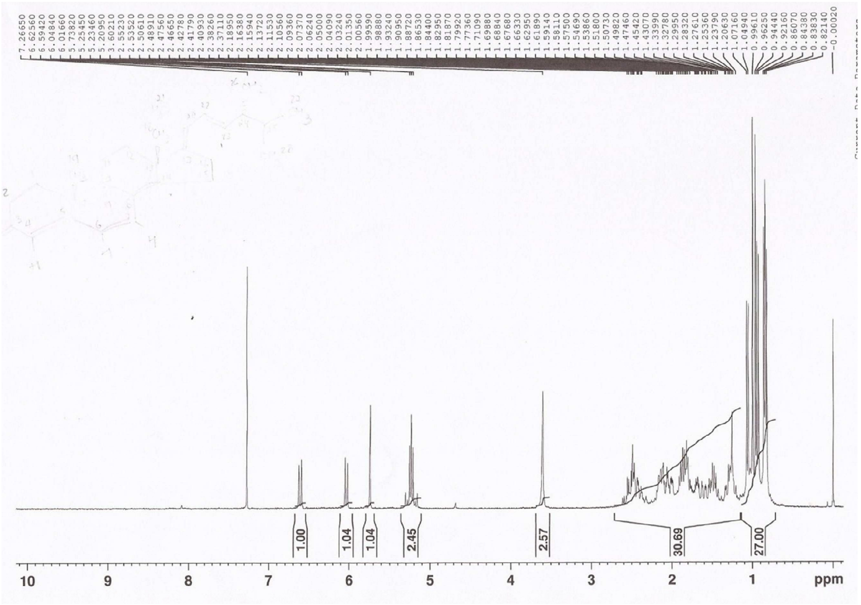

Figure S21. ^{13}C NMR spectrum of ergosta-4,6,8 (14), 22-tetraen-3-one ($CDCl_3$, 75.4 MHz).

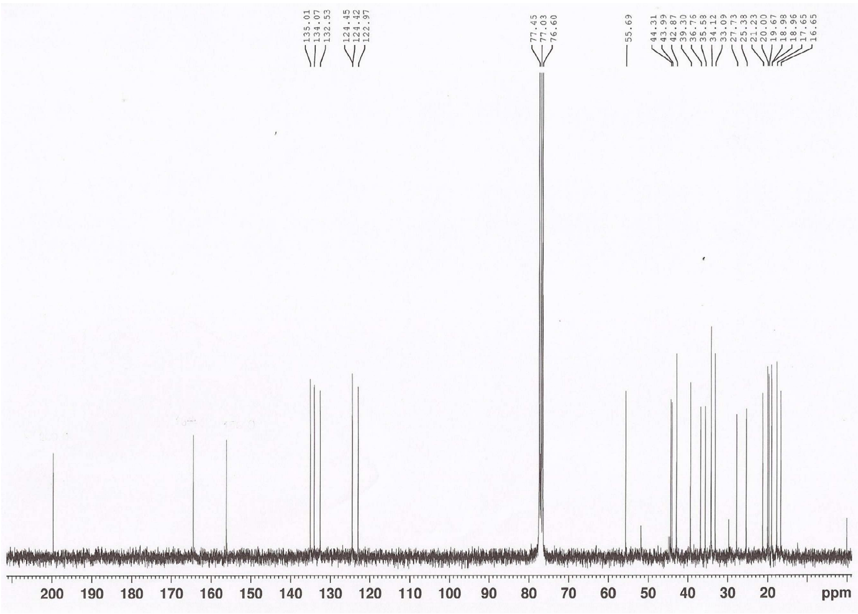

Figure S22. ^{1}H NMR spectrum of cyathisterone ($CDCl_3$, 300.13 MHz).

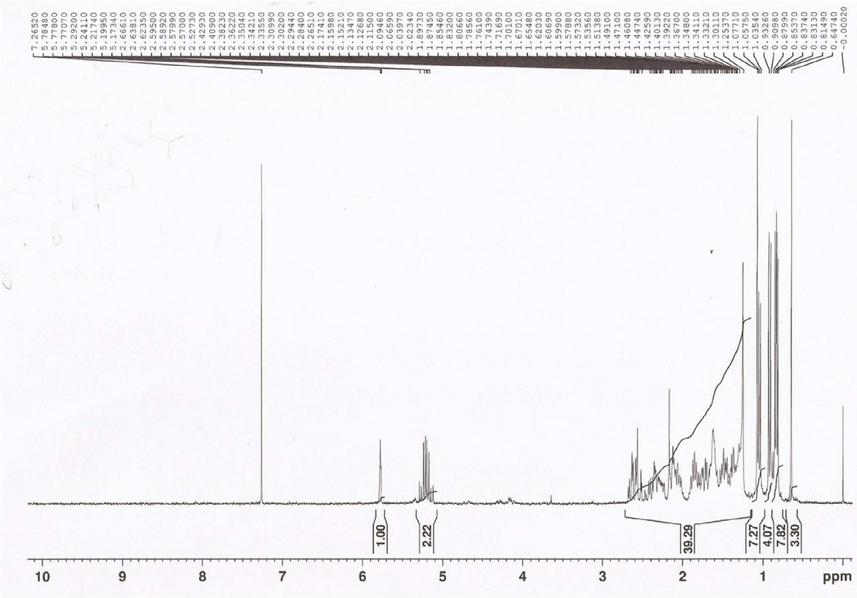

Figure S23. ^{13}C NMR spectrum of cyathisterone ($CDCl_3$, 75.4 MHz).

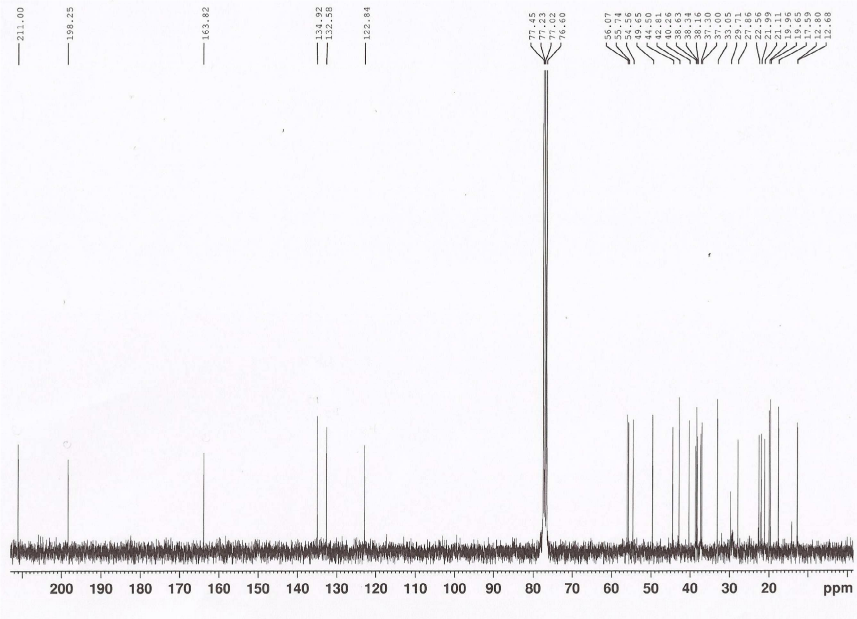

Figure S24. ^{1}H NMR spectrum of dehydromevalonic acid lactone ($CDCl_3$, 300.13 MHz).

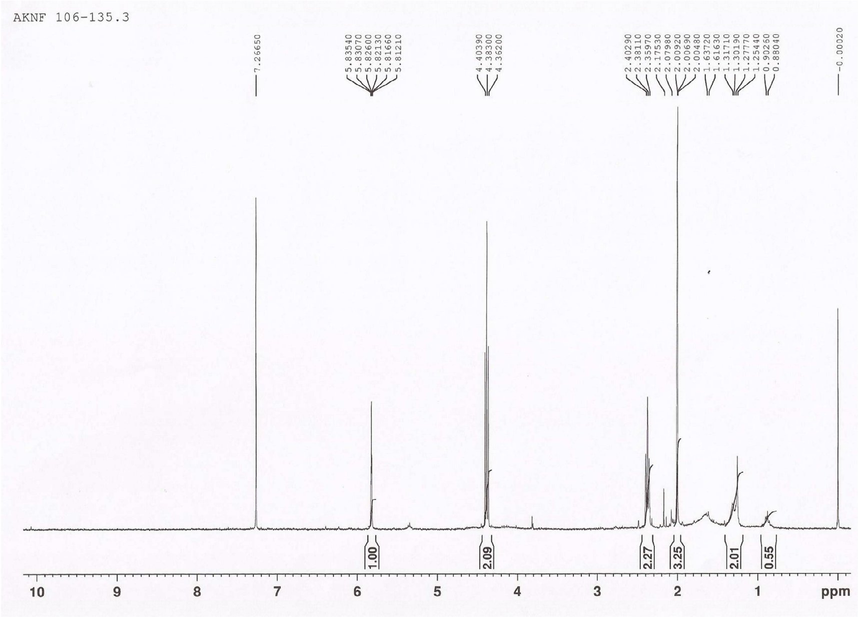

Figure S25. ^{13}C NMR spectrum of dehydromevalonic acid lactone ($CDCl_3$, 75.4 MHz).

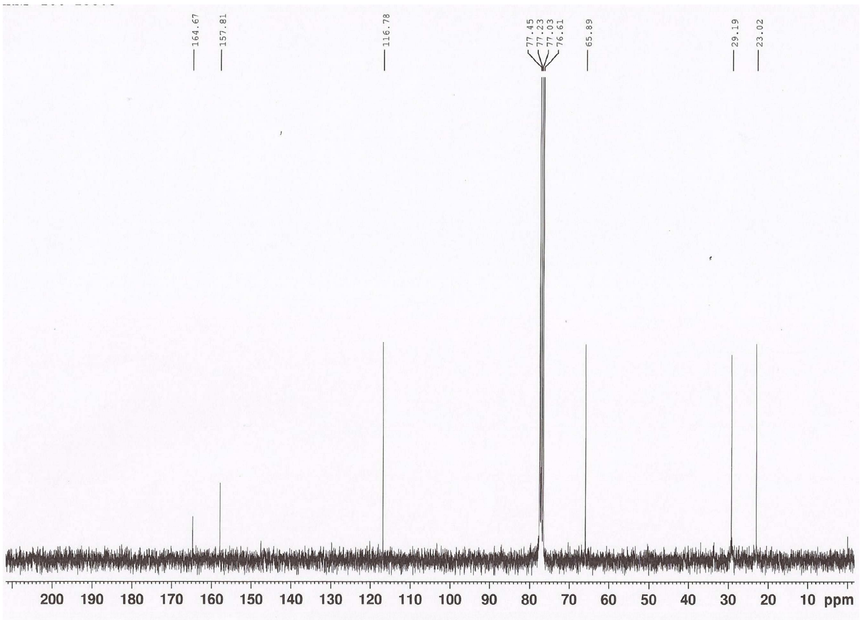

Figure S26. ^{1}H NMR spectrum of aszonalenin ($CDCl_3$, 300.13 MHz).

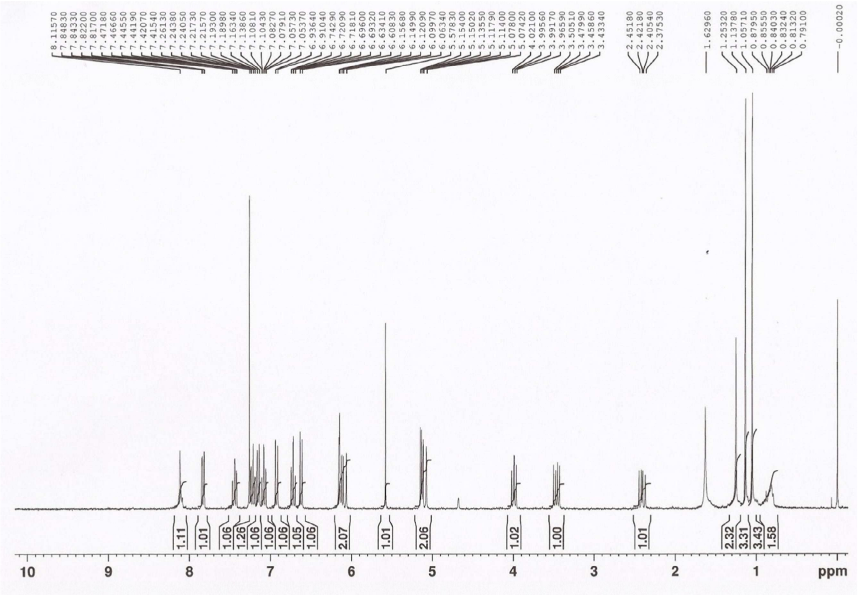

Figure S27. ^{1}H NMR spectrum of aszonalenin ($CDCl_3$, 75.4 MHz).

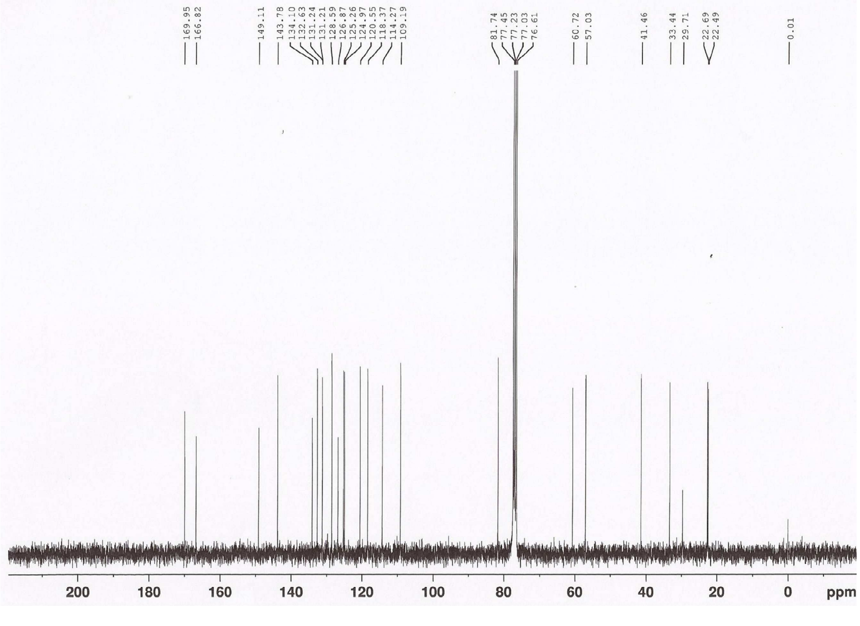

Figure S28. ^{1}H NMR spectrum of secalonic acid A ($CDCl_3$, 300.13 MHz).

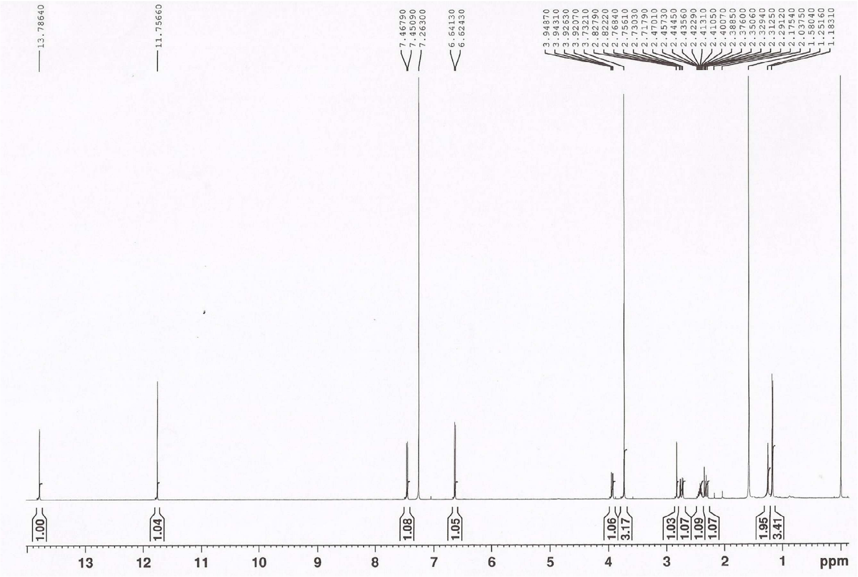

Figure S29. ^{13}C NMR spectrum of secalonic acid A ($CDCl_3$, 75.4 MHz).

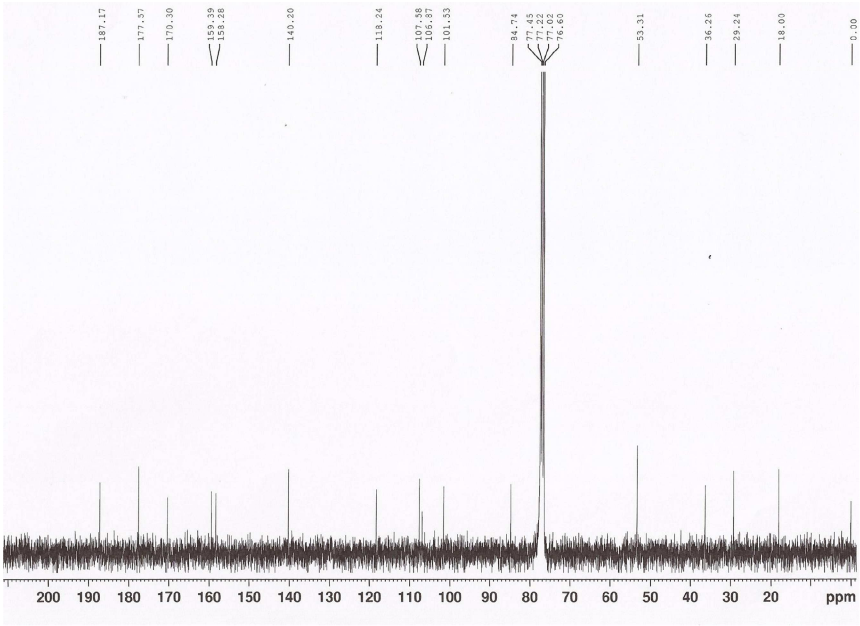

Figure S30. ^{1}H NMR spectrum of fellutanine A (DMSO, 300.13 MHz).

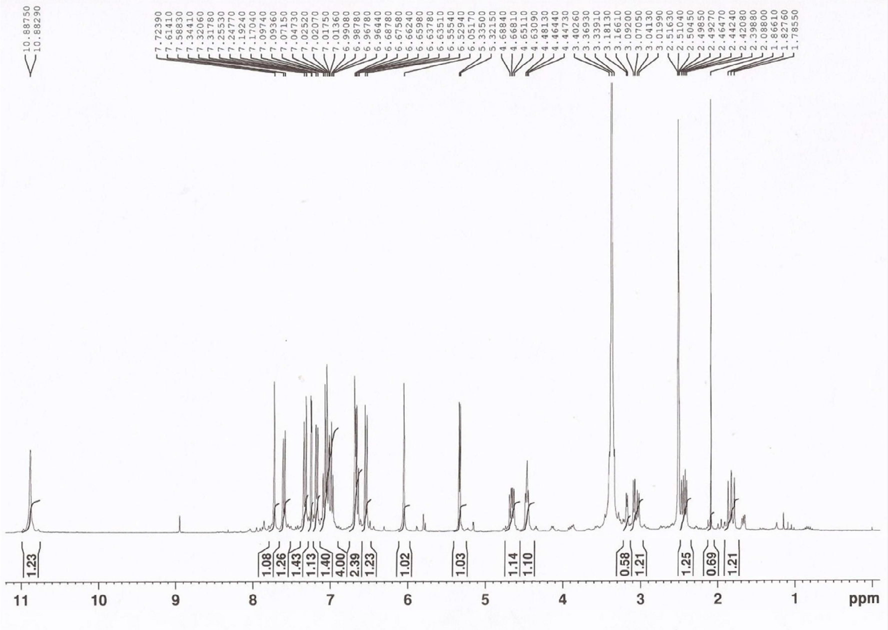

Figure S31. ^{13}C NMR spectrum of fellutanine A (DMSO, 75.4 MHz).

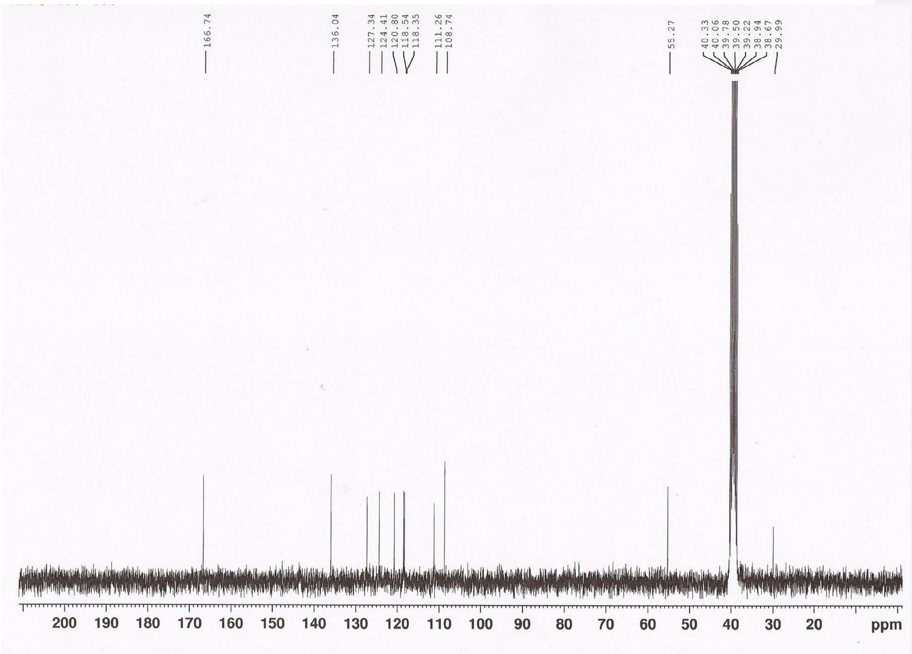

Figure S32. ^{1}H NMR spectrum of **1** (DMSO, 500.13 MHz).

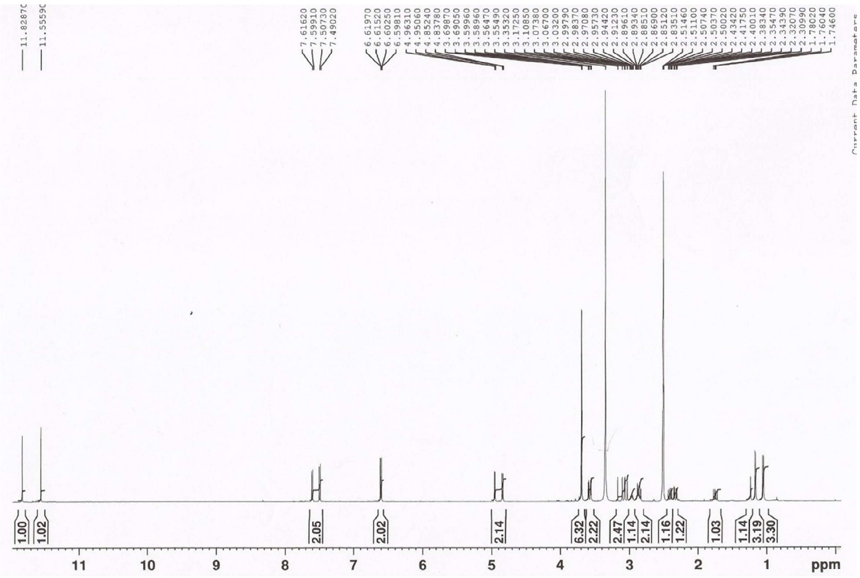

Figure S33. ^{13}C NMR spectrum of **1** (DMSO, 125.8 MHz).

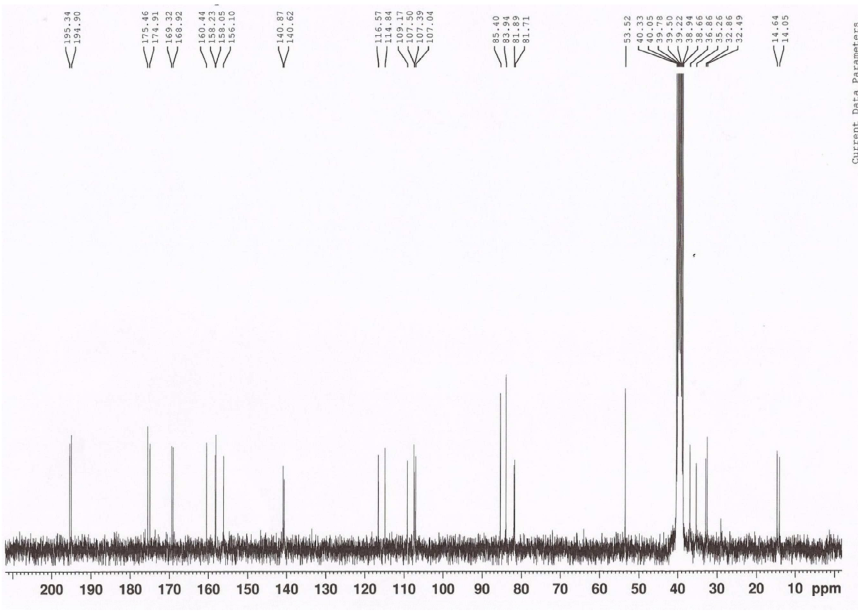

Figure S34. COSY spectrum of **1** (DMSO, 500.13 MHz).

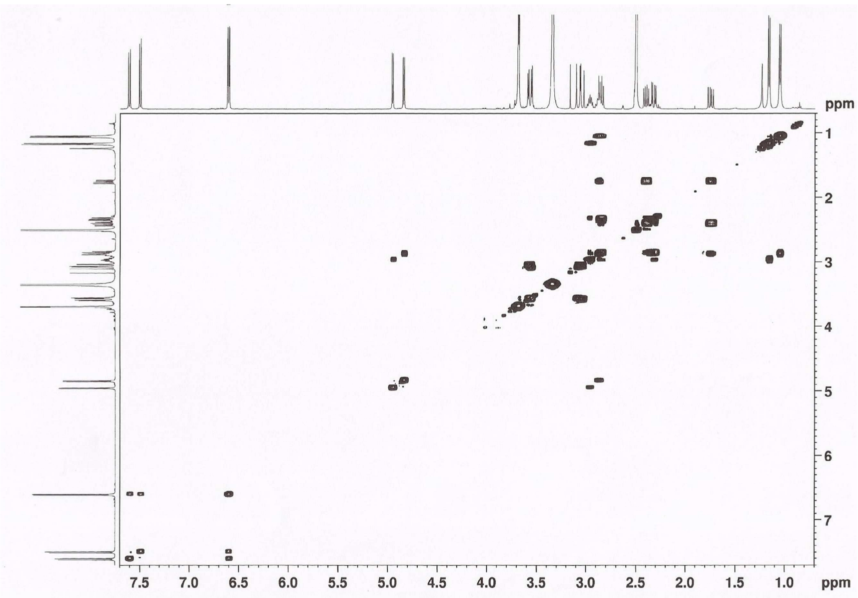

Figure S35. HSQC spectrum of **1** (DMSO, 500.13 MHz).

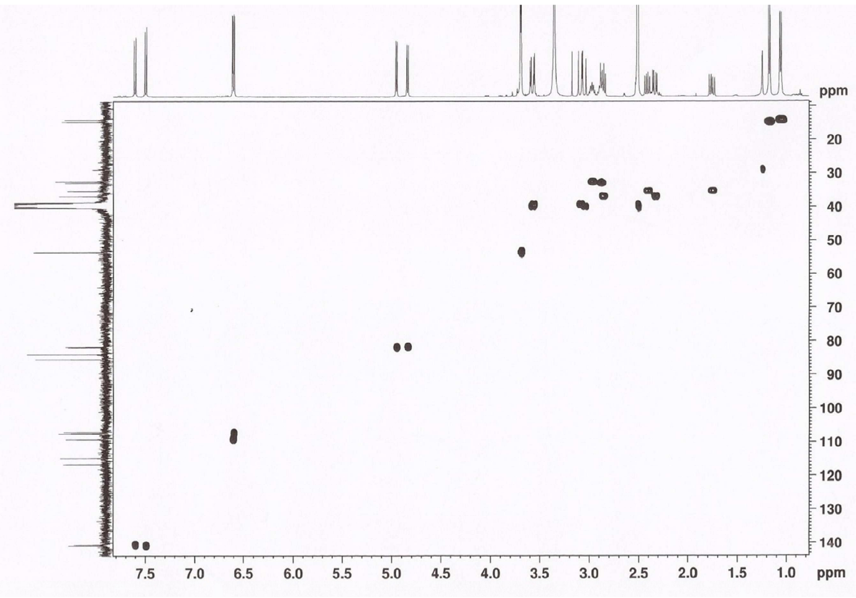

Figure S36. HMBC spectrum of **1** (DMSO, 500.13 MHz).

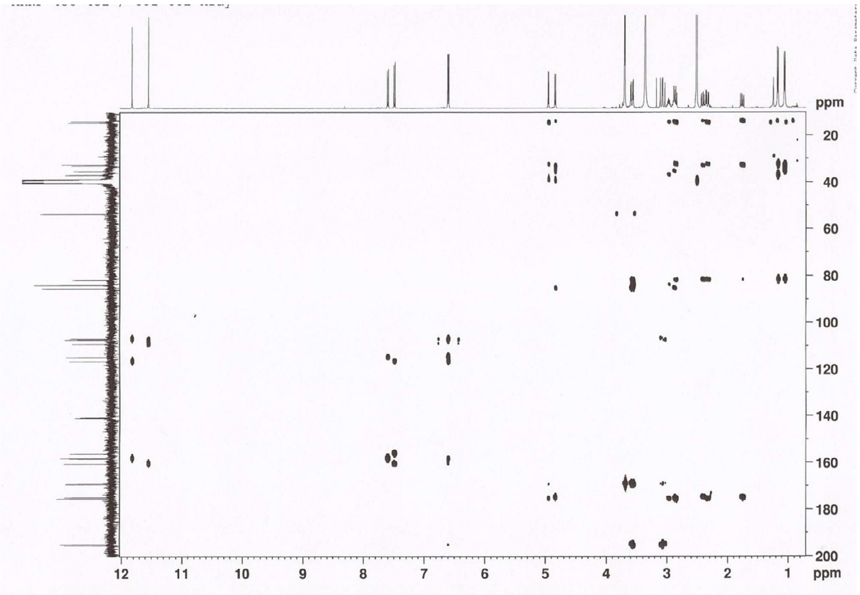

Figure S37. ^{1}H NMR spectrum of **2** ($CDCl_3$, 500.13 MHz).

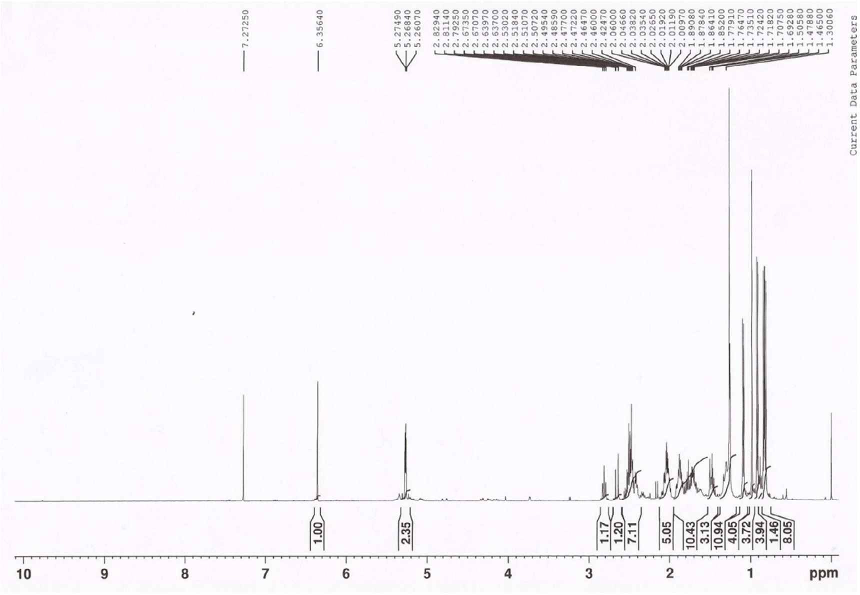

Figure S38. ^{13}C NMR spectrum of **2** ($CDCl_3$, 125.8 MHz).

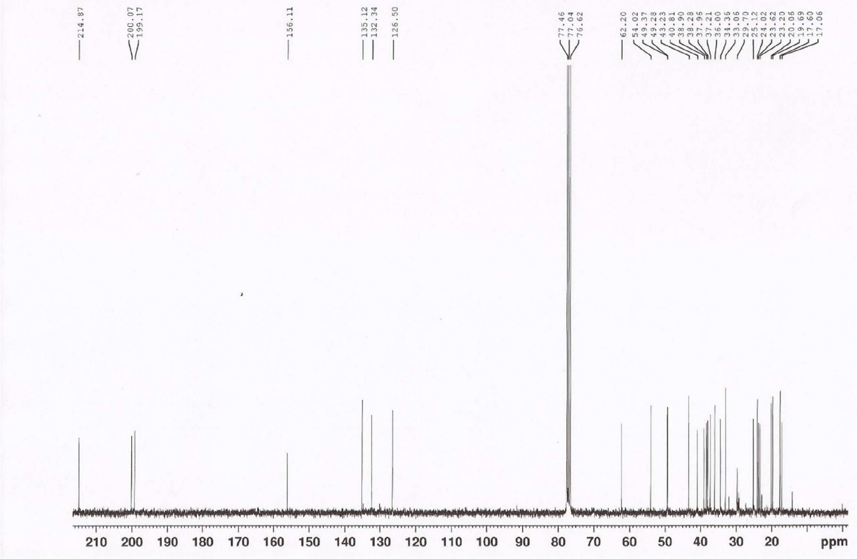

Figure S39. HSQC spectrum of **2** ($CDCl_3$, 125.8 MHz).

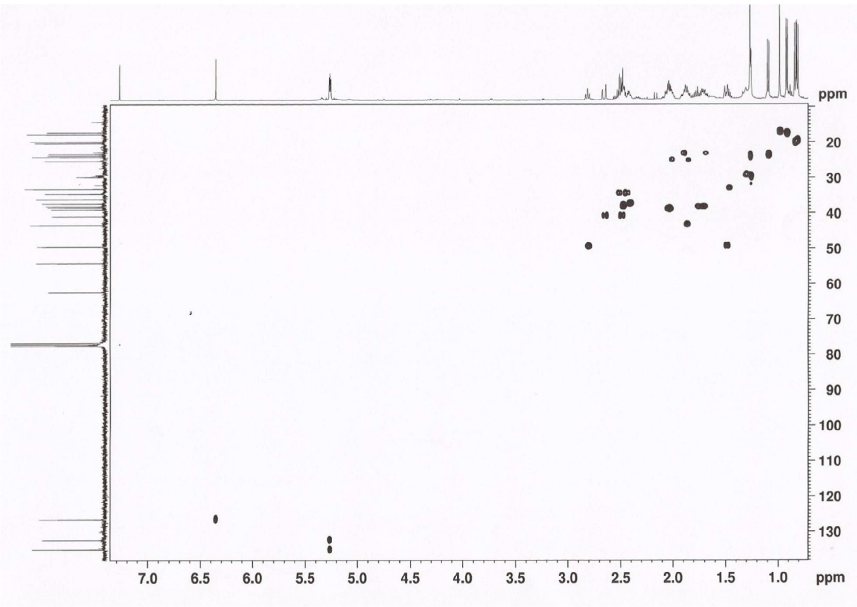

Figure S40. HMBC spectrum of **2** ($CDCl_3$, 125.8 MHz).

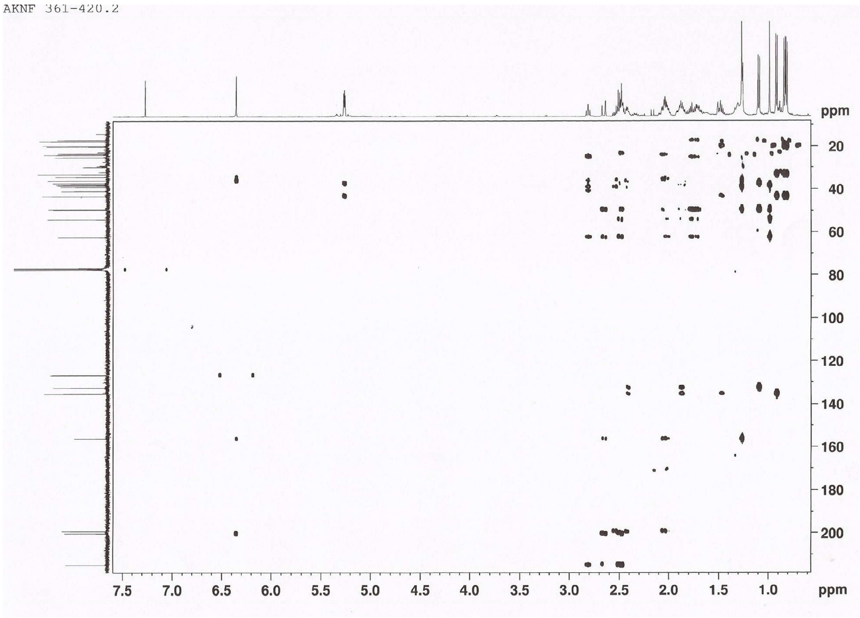

Figure S41. ^{1}H NMR spectrum of **3** ($CDCl_3$, 300.13 MHz).

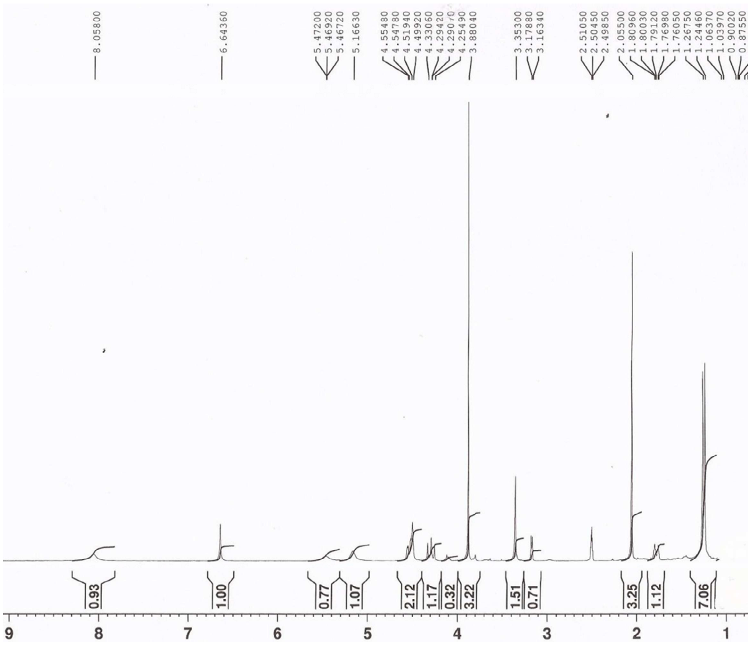

Figure S42. ^{13}C NMR spectrum of **3** ($CDCl_3$, 75.4 MHz).

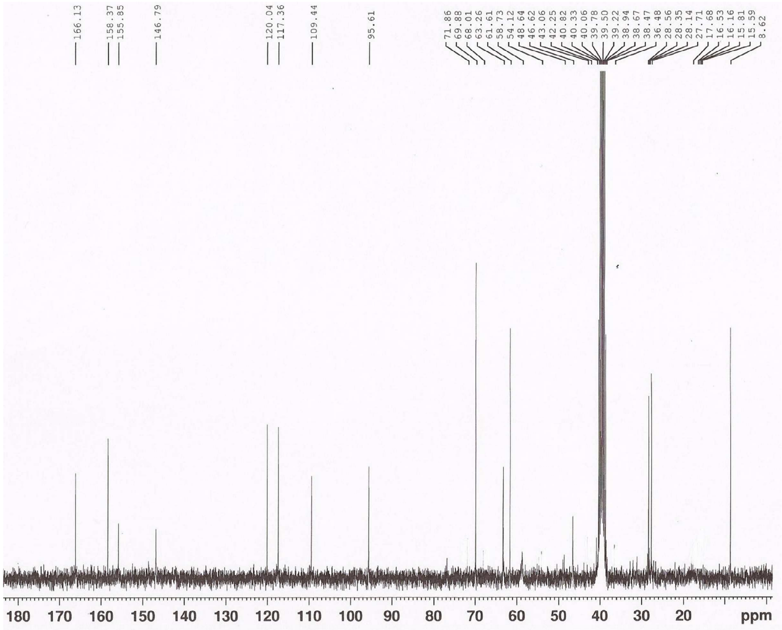

Figure S43. COSY spectrum of **3** ($CDCl_3$, 300.13 MHz).

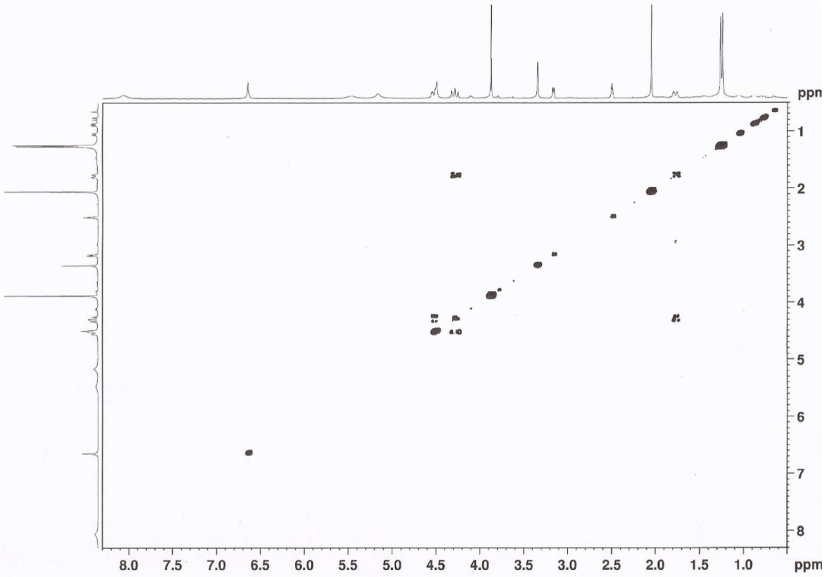

Figure S44. HSQC spectrum of **3** ($CDCl_3$, 300.13 MHz).

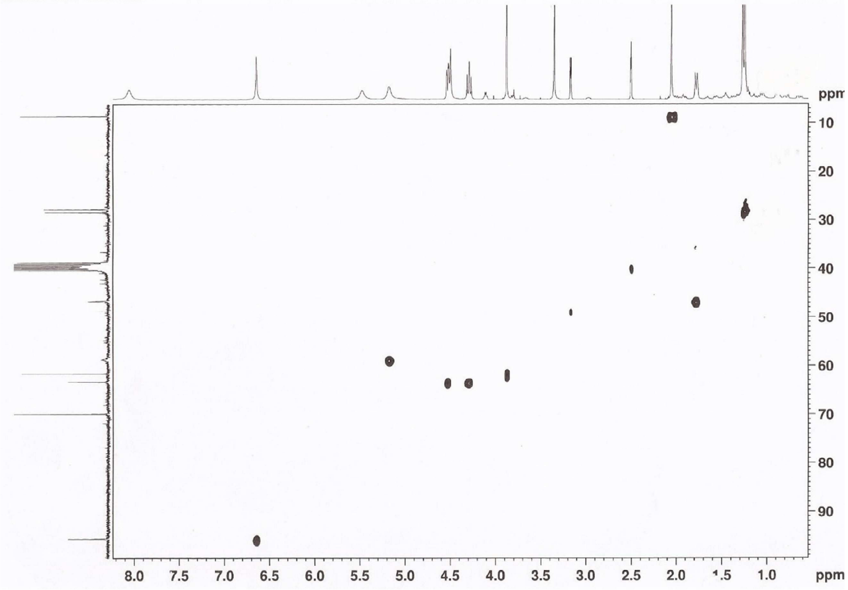

Figure S45. HMBC spectrum of **3** ($CDCl_3$, 300.13 MHz).

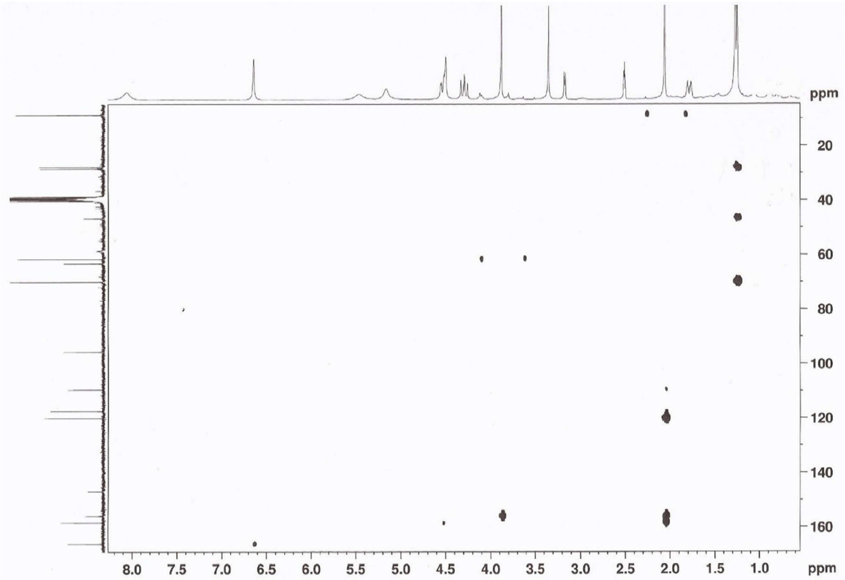

Figure S46. NOESY spectrum of **3** ($CDCl_3$, 300.13 MHz).

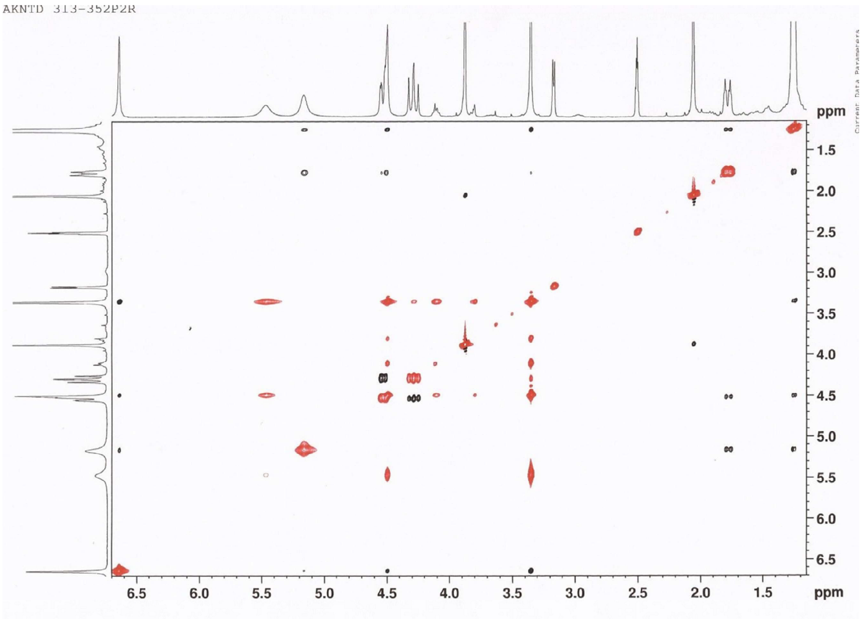

Figure S47. ^{1}H NMR spectrum of **4** ($CDCl_3$, 500.13 MHz).

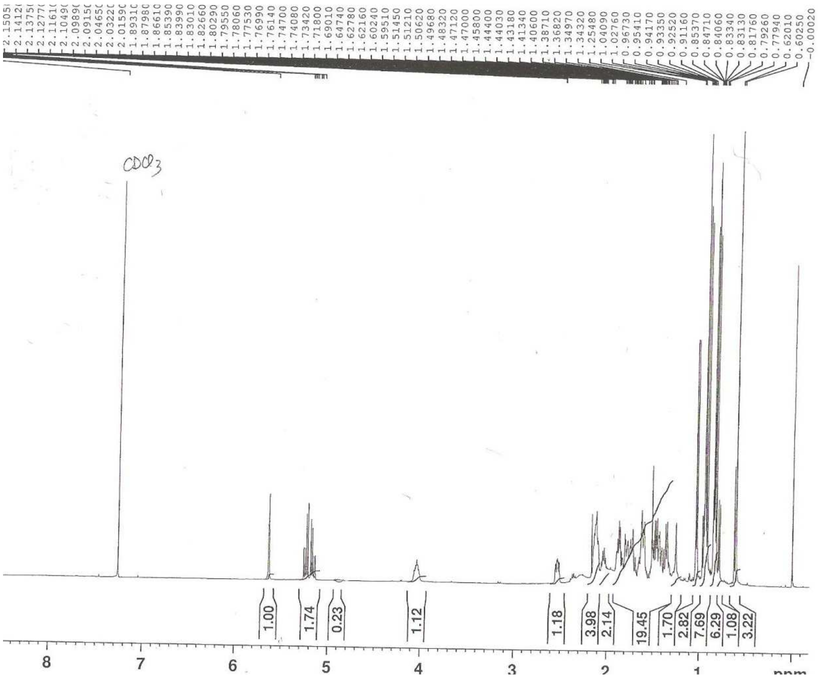

Figure 48. ^{13}C NMR spectrum of **4** ($CDCl_3$, 125.4 MHz).

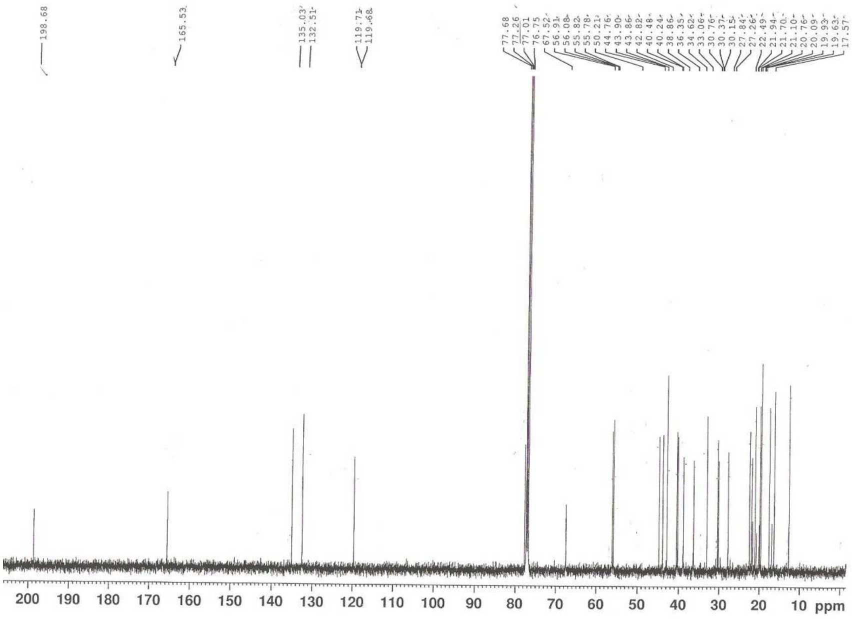

Figure S49. Ortep view of dankasterone A (**2**)

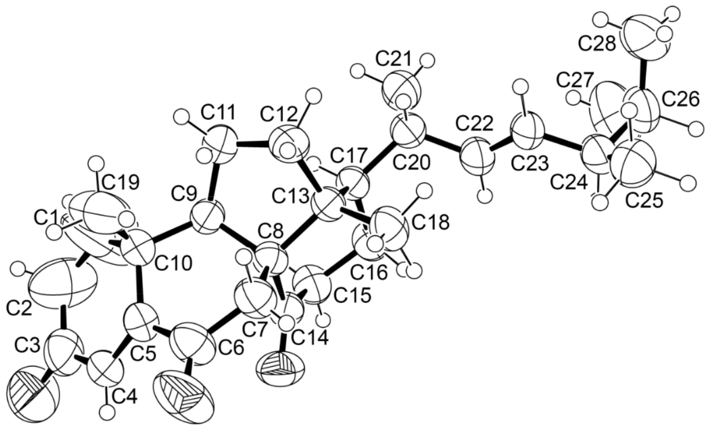

Table S1

^{1}H and ^{13}C NMR ($CDCl_3$, 500 MHz and 125 MHz) and HMBC assignment for **2**

Position	δ_C, type	δ_H, (*J* in Hz)	COSY	HMBC
1	38.9, CH_2	2.05, m	H-2	C-3, 5
2	34.4, CH_2	2.46, m	H-1	
3	199.2, CO	-		
4	126.5, CH	6.36, s	-	C-2, 5, 6, 10
5	156.1, C	-		
6	200.1, CO	-		
7α	40.8, CH_2	2.49, d (16.9)	H-7β	C-6, 8, 13
β		2.66, dd (16.9, 1.4)	H-7α	C-5, 6, 8, 9
8	62.2, C	-		
9	49.3, CH	2.81, brt (9.0)	H-11	C-1, 7, 8, 10, 14, 19
10	36.0, C	-		
11	23.2, CH_2	1.68, m	H-9, 12	
		1.88, m	H-9, 12	
12	38.3, CH_2	1.71, m	H-11	
		1.76, m	H-11	
13	54.0, C	-		
14	214.9, CO	-		
15	38.0, CH_2	2.47, m	H-16	C-14, 17
16	25.1 CH_2	1.85, m	H-15, 17	
		2.05, m	H-15, 17	
17	49.4, CH	1.48, m	H-16, 20	
18	17.1, CH_3	0.98, s	-	C-8, 12, 13,
19	24.0, CH_3	1.26, s	-	C-1, 5, 9, 10
20	37.2, CH	2.41, m	H-17, 21, 22	C-22, 23
21	23.6, CH_3	1.09, d (7.0)	H-20	C-17, 20, 23
22	135.1, CH	5.27, m	H-20, 23	C-24
23	132.3, CH	527, m	H-22, 24	C-20
24	43.2, CH	1.87, m	H-23, 26	
25	33.1, CH	1.46, m	H-24, 27, 28	
26	17.6, CH_3	0.91, d (6.9)	H-24	C-22, 24, 25
27	20.1, CH_3	0.83, d (6.8)	H-25	C-24, 25, 28
28	19.7, CH_3	0.81, d (6.8)	H-25	C-24, 25, 27

Table S2

^{1}H and ^{13}C NMR ($CDCl_3$, 500 MHz and 125 MHz) of **4.**

Position	δ_C, type	δ_H, (*J* in Hz)	HMBC

1	27.8, CH_2	1.35, m<br>1.77, m	
2	30.4, CH_2	1.61, m<br>1.79, m	
3	67.5, CH	4.04, q (6.4)	
4	36.4, CH2	1.61, m<br>1.79, m	
5	77.7, C	-	
6	198.7, CO	-	
7	119.7, CH	5.63, brs	C-5, 9, 14
8	165.5, C	-	
9	43.9, CH	2.53, ddd (11.8, 7.9, 2.2)	C-7, 8, 10, 11, 19
10	40.5, C	-	
11	21.9, CH_2	1.61, m<br>1.72, m	
12	38.9, CH_2	1.43, m<br>2.10, m	
13	44.8, C	-	
14	55.8, CH	2.13, m	
15	22-5, CH_2	1.35, m<br>1.77, m	
16	30.2, CH_2	1.42, m<br>1.87, m	
17	56.1, CH	1.35, m	
18	12.7, CH_3	0.60, s	C-12, 13, 17
19	16.4, CH_3	0.94, s	C-1, 5, 7
20	40.2, CH	2.03, m	
21	21.1, CH_3	1.03, d (6.6)	C-17, 20
22	135.0, CH	5.16, dd (15.3, .6)	C-17, 20, 21, 24
23	132.5, CH	5.24, dd (15.3, 7.6)	C-20, 24, 25, 26
24	42.8, CH	2.03, m	
25	33.1, CH	1.47, m	
26	17.6, CH_3	0.92, d (6.8)	C-23, 24, 25
27	19.9, CH_3	0.84, d (6.9)	C-24, 25, 28
28	19.6, CH_3	0.82, d (6.9)	C-24, 25, 27
